# Novel insights into ORFV B2L DNA vaccine-mediated gut microbiota modulation and immune augmentation in rats

**DOI:** 10.3389/fimmu.2025.1598969

**Published:** 2025-07-18

**Authors:** Umar Farooq, Guiqiong Liu, Sohail Ahmed, Huiguo Yang, Mehboob Ahmed, Xunping Jiang

**Affiliations:** ^1^ Key Laboratory of Agricultural Animal Genetics, Breeding and Reproduction of Ministry of Education, Huazhong Agricultural University, Wuhan, China; ^2^ Laboratory of Small Ruminant Genetics, Breeding and Reproduction, College of Animal Science and Technology, Huazhong Agricultural University, Wuhan, China; ^3^ Department of Animal Science Research Institute, Xinjiang Academy of Animal Sciences, Urumqi, China

**Keywords:** Orf virus, DNA vaccine, gut microbiota, immune response, vaccine development

## Abstract

The Orf virus (ORFV) poses a significant threat to livestock and human health, causing economic losses in the livestock industry and potential zoonotic infections. Given the limitations of current vaccines, the objective of this study was to investigate the immune response and gut microbiota modulation induced by the ORFV B2L gene-based DNA vaccine (GV) and the live attenuated vaccine (LV) in rats. The findings of this study will provide a scientific foundation for the development of more effective vaccines. Female Sprague-Dawley rats, which were free of specific pathogens, were divided into three groups. The experiment included three groups: the first group was designated as the GV group, the second group was designated as the LV group, and the third group was designated as the control group. Rats in the GV group received intra-muscular injection of 100μg/dose of pVAX - B2L - asd plasmid, those in the LV group were immunized with a commercial live - attenuated vaccine, and the control group was injected with PBS. After immunization, various immune - related parameters, such as T - cell subsets, antibody levels, cytokines, and oxidative stress markers, were measured. To this end, composition and function of gut microbiota were thoroughly examined through the implementation of 16S rRNA gene sequencing and PICRUSt-2 functional prediction. The GV group exhibited elevated levels of cellular and humoral immunity. It had a higher percentage of CD4^+^ and CD8^+^ T cells, enhanced levels of cytokines i.e. IL - 2, IL - 6, and TNF - α, elevated IgA, IgG antibody production compared to the LV and control groups. Additionally, the GV group showed reduced oxidative stress. In terms of gut microbiota, GV immunization led to an increase in beneficial bacteria like Lachnospi-raceae_NK4A136_group and a decrease in harmful or potentially pathogenic bacteria. KEGG pathway analysis indicated that differential flora exhibited an increase in metabolic pathway diversity, including those related to biological systems, metabolism, and human diseases. In sum, the results of the present study demonstrate that the ORFV B2L DNA vaccine (GV) elicited a more robust immune response and exerted a beneficial effect on composition and function of the gut microbiota compared with ORF live-attenuated vaccine. The results of the present study indicate that modulation of gut microbiota by GV vaccine play a crucial role in enhancing vaccine efficacy. The current study provides new perspectives on ORFV vaccine development and its association with vaccines and gut microbiota modulation.

## 1 Introduction

The ORF virus (ORFV), characterized by an enveloped structure and a double-stranded DNA genome ranging from 132 to 140 kb and encoding 132 genes, belongs to the family Poxviridae and subfamily Chordopoxvirinae of the genus Parapoxvirus. It causes contagious pustular dermatitis and poses a serious zoonotic concern, leading to substantial economic losses in livestock production (1). The ORFV infection causes organ-specific lesions in lambs, affecting oral cavity, lips, labial commissure, nasals, dental pad, gums, tongue, eyelids, teeth, feet, esophagus, stomach, intestine, and respiratory tract (2). Despite its low mortality rate, ORFV infection results in considerable economic losses in lambs due to difficulties in suckling or eating caused by oral lesions and associated secondary infections (3).

Moreover, the high morbidity rate, slow growth, and culling of seriously infected lambs and kids adversely affect small ruminant farm production and the income of rural com-munities reliant on livestock farming (3). ORFV encodes various immunomodulatory proteins that evade host immune responses and may lead to repeated infections in animals. Multiple outbreaks of ORFV infection have been reported globally in recent years. Although licensed live-attenuated and commercially available vaccines exist, they fail to confer effective immunity against the disease (4).

Gut microbiota has been reported to modulate immune responses to various vaccines (5), playing a vital role in regulating and developing immune responses, as well as maintaining balance of highly specialized immune cells in the host (6–8). Also, it can stimulate both innate and adaptive immune responses, which are crucial for effective vaccine function (9). On the other hand, while the host immune response to vaccines has been extensively studied across various aspects of the immune system, the relationship be-tween host immune responses and gut microbiota dynamics remains unclear. The maintenance of a robust and healthy gut microbiota is of paramount importance, as it plays a pivotal role in safeguarding the integrity of the mucosal barrier. This barrier, which serves as the initial line of defense against potential pathogens, is crucial for maintaining optimal health and well-being (10). Therefore, disruption of mucosal barrier by pathogens can alter the immune response to vaccines and make the holistic evaluation of vaccine safety questionable. However, increasing evidence suggests that a healthy, intact gut microbiota and its products play essential roles in maintaining host homeostasis (11).

The microbiota has been demonstrated to produce a variety of metabolites, including short-chain fatty acids (SCFAs). These metabolites have the capacity to influence the function of immune cells, thereby potentially modulating the responses to vaccines (12). Consequently, composition of gut microbiota may exert pivotal effect on vaccines effectiveness. Gut microbiota has been shown to interact with each other through various physiological mechanisms. These mechanisms include the generation of metabolites that activate lymphocytes or antigen-presenting cells, the activation of the inflammasome in innate cell populations, and the direct action on epithelial cells. These interactions ultimately control the immune response (13). As indicated by GALT, composition of gut microbiota is also critical to maturation of immune system, particularly in gut-associated lymphoid tissue (14). This is particularly important as alterations in commensals can influence susceptibility to gastrointestinal diseases, and vaccine efficacy (15). Gut microbiota is known to interact with immune system of host exerts a significant influence on its development and function (16). These interactions may have both positive and negative effects on overall health and well-being (14). The immunogenicity of vaccines, which relies on the host’s ability to generate a robust and specific immune response, is, therefore, likely to be affected by the gut microbiota composition (17).

Moreover, immune response dysfunction and gut microbiota alteration involve both innate and adaptive immune responses, with a strong association between these two vital systems. Gut microbiota primarily modulates immune responses, nutrient exchange, gut morphology, detoxification, and protection against pathogens, ultimately influencing the health and growth performance of animals (18, 19). A balanced microbiota is essential for the proper education of immune system, which can affect and modulate vaccination response.

However, to the best of our knowledge, the impact of ORF - GV vaccine and ORF - LV on the host gut microbiota remains uncharted territory. It is imperative to acknowledge the pivotal function of gut microbiota in regulating immune responses, maintaining mucosal barrier integrity, and influencing vaccine efficacy, understanding how these vaccines interact with the gut microbiota is of paramount importance. ORFV continues to be a persistent threat to livestock and public health, and current vaccination strategies have significant drawbacks. Live - attenuated vaccines, although available, often fail to provide long - lasting immunity and may even pose risks such as reversion to virulence. DNA vaccines, on the other hand, hold promise but their interaction with gut microbiota, a key player in immune system, has not been explored.

This knowledge gap hinders the development of more effective vaccines against ORFV. Therefore, present study was designed to fill this void. By utilizing a rat model, we aimed to comprehensively investigate the immune responses induced by ORF - GV and ORF - LV vaccines and their potential associations with gut microbiota modulation be-fore and after immunization. The development of subcutaneous immunization models, combined with 16S rRNA sequencing of the gut microbiome, enabled a comparative examination of the differences in gut microbial composition across various groups. The findings of this investigation are anticipated to furnish a robust basis for the advancement of enhanced vaccines against ORF virus, thereby offering fresh perspectives on the intricate interrelationship between vaccines and the intestinal microbiota. This advancement holds the potential to bring about a fundamental transformation in the domain of ORFV vaccination.

## 2 Materials and methods

### 2.1 Experimental animals

Female Sprague-Dawley (SD) rats, 6 weeks old and weighing approximately 200 grams, were sourced from Wuhan Mobaili Biotechnology Co., Ltd. These rats were certified to be free of specific pathogens. They were housed in the Laboratory Animal Centre at Huazhong Agricultural University, China, under controlled conditions. The animals were kept in a pathogen-free environment with a 12-hour light/dark cycle, ambient humidity of 50–60%, and a temperature maintained at around 22°C. During the experimental period, the rats were provided with sterilized food and water ad libitum. Prior to inclusion in the study, the rats underwent a two-week acclimatization period to ensure adaptation to their new environment and minimize stress-related variables.

### 2.2 Ethical statement

The study was approved by the Institutional Animal Care and Use Committee (IACUC) of Huazhong Agricultural University, Wuhan, China (HZAUGO-2019–006). All procedures were conducted in accordance with the ethical guidelines for the care and use of laboratory animals.

### 2.3 Experimental setup and immunization procedure

The experiment was designed using a completely randomized method, with thirty six rats being equally allocated to one of three groups. (A) ORFV B2L gene-based DNA (ORF-GV) group, where rats received 100μg/dose of pVAX-B2L-asd plasmid diluted in 1ml saline via intramuscular injection. (B) ORF live attenuated (ORF-LV) group, where rats were immunized with commercially available ORFV live attenuated vaccine at a dose of 0.1 ml (>10×5 TCID50/0.1). The vaccine used was Yang Chuanping (HCE Zhu), an infectious pustular dermatitis vaccine derived from the live HCE strain, manufactured by Shandong Huahong Biological Engineering Co., Ltd., with approval number veterinary drug Nweward 150104032, and administered in accordance with the manufacturer’s prescribed guidelines. (C) Control (CT) group, in which rats received a placebo injection of PBS at a volume of 1 ml via intramuscular injection. The 2nd booster dose of respective vaccine was administered 4-weeks after the initial immunization.

### 2.4 Sample collection

Experimental rats were euthanized via cervical dislocation in accordance with established ethical guidelines and approved protocols (20), and the spleen and gastrointestinal tract were carefully isolated. Subsequently, the intestinal contents from the caecum were collected in sterile tubes and promptly stored at -80°C to maintain the integrity of the samples until further analysis. The caecal contents of the rats were collected both before and after immunization, precisely at 28 days and 60 days after the second booster immunization.

### 2.5 Immunophenotyping of CD4^+^ and CD8^+^ T cells (flow cytometry assay)

The immunophenotyping of T-cell subsets (CD4^+^ and CD8^+^) was conducted by adjusting the spleen cells to a concentration of 1 x 10^7^ cells/mL, in accordance with the method previously outlined (20). The red blood cells were lysed with erythrocyte lysate (Solarbio, Beijing, China) and subsequently washed with phosphate-buffered saline (PBS) three times. The cells were stained with allophycocyanin (APC)-conjugated anti-rat CD8^+^ (Intervengen), fluorescein isothiocyanate (FITC)-conjugated anti-rat CD4^+^, and phycoerythrin (PE)-conjugated anti-rat CD3^+^ (Wuhan Saituobaiao Bioengineering Co., Ltd.) at 4°C for 30 minutes. The antibodies-stained cells underwent a thorough washing process with cell staining buffer (BioLegend, San Diego, CA, USA) to ensure optimal staining removal. Following this, the cells were resuspended in 0.5 mL of flow buffer, a crucial step in preparing the samples for analysis by a flow cytometer (Syntax) utilizing FlowJo software (Tree Star, Ashland, OR, USA).

### 2.6 Assessment of antibody responses, immunoglobulin isotypes, secretory IgA, and cytokine levels

The determination of serum anti-B2L antibody was accomplished through the implementation of indirect enzyme-linked immunosorbent assay (ELISA) technique in accordance with established references (21). The plates utilized in this study were synthetic B2L antigen-coated plates manufactured by Bioss (Beijing, China). An HRP-goat anti-rat IgG secondary antibody (Bioss, Beijing, China) at a dilution of 15,000 was employed to detect specific antibodies. The final enzymatic reaction was expressed as an antibody titre and determined at an OD of 450 nm. An ELISA procedure analogous to the one previously described was employed to assess levels of IgA, IgG, and IgM in serum. This ELISA procedure was carried out using indirect ELISA kits manufactured by Shanghai Enzyme Linked Biotechnology Co., Ltd. The sIgA content present within the homogenized samples derived from the duodenum, jejunum, ileum and caecum was measured using a sIgA Enzyme-Linked Immunosorbent Assay (ELISA) kit (product number YJ003136) manufactured by Beijing Solarbio Science & Technology Co., Ltd., located in Beijing, China. The procedure was carried out in strict accordance with the manufacturer’s guidelines. The degree of light absorption was measured at a wavelength of 450 nanometers (OD), and the concentration of sIgA was calculated based on a standard curve. Furthermore, the levels of serum cytokine responses were determined in rats’ serum using ELISA Kits from Jianglai Bio (Shanghai, China) following the manufacturer’s instructions.

### 2.7 Analysis of redox status biomarkers

An analysis was conducted of the levels of malondialdehyde (MDA), glutathione peroxidase (GPX), superoxide dismutase (SOD), and catalase (CAT) in the serum of immunized rats. These samples were obtained before and after the use of commercial assay kits (Nanjing Institute of Jiancheng Biological Engineering, Nanjing, China), in accordance with the manufacturer’s instructions. This analysis was performed as previously described in a related study (22, 23).

### 2.8 Sample preparation and library sequencing

The fecal contents remained thawed during storage until transfer to Wuhan. The company under discussion here is IGENEBOOK Biotechnology Co., Ltd., a business headquartered in Wuhan, China (see http://www.igenebook.com). The company specializes in microbiome sequencing.

### 2.9 Microbial DNA extraction

The isolation of microbial DNA from the caecal contents was performed using a microbial genomic DNA extraction kit (Guangzhou Magen Biotechnology Co., Ltd, Guangzhou, China), specifically Item # D314103D, in accordance with the manufacturer’s protocols. Subsequently, the final DNA concentration and purity were determined using the Nano Drop 2000 UV-vis spectrophotometer (Thermo Scientific, Wilmington, Delaware, United States). DNA quality was then assessed through 2% agarose gel electrophoresis (24). To further analyze the samples, V3-V4 hyper variable regions of the bacterial 16S rRNA gene were amplified using the primers 338F (5’-ACTCCTACGGGAGGCAGCAG-3’) and 806R (5’-GACTACHVGGGTWTCTAAT-3’) via thermo cycler PCR system (Gene Amp 9700, ABI, Foster, CA, United States). Subsequent to amplification, the extracted DNA was stored at −80°C for subsequent analysis.

### 2.10 PCR amplification

The thermal cycling protocol comprised the following steps: denaturation at 95°C for 3 minutes, 27 cycles of denaturation at 95°C for 30 seconds, annealing at 55°C for 30 seconds, elongation at 72°C for 45 seconds, extension at 72°C for 10 minutes, and ending at 4°C. Each 20 μL reaction mixture contained 4 μL of 5× Trans Start FastPfu buffer, 2 μL of 2.5 mM dNTPs, 0.8 μL of each primer (5 μM), 0.4 μL of Trans Start FastPfu DNA Polymerase, and 10 ng of template DNA. The PCR was performed in triplicate. The PCR product was extracted from a 2% agarose gel and purified using an AxyPrep DNA Gel Extraction Kit (Axygen Biosciences, Union City, CA), according to the manufacturer’s instructions, and quantified using Qubit 4 (Thermo Fisher, United States).

### 2.11 16S ribosomal RNA gene sequencing

Subsequent purification of the amplicons was conducted, followed by the pooling of equimolar amounts and the subsequent subjection of the amplicons to paired-end sequencing. This sequencing was performed using the Illumina MiSeq PE300 platform (Illumina, San Diego, United States). The standard protocols provided by Honsunbio Technology Co. were utilized for this sequencing. Ltd (Shanghai, China) were followed.

### 2.12 Bioinformatics analysis

The sequencing reads underwent several pre-processing steps to ensure data quality and reliability. Initially, demultiplexing and quality control were performed using fastp (version 0.21.0), followed by adapter sequence removal and trimming of low-quality bases (quality score <Q 20) (25). Reads that were shorter than 50 base pairs in length, as well as those containing ambiguous nucleotides, were excluded from further analysis.

Subsequently, the paired-end reads were merged using FLASH (version 1.2.7), with a minimum overlap of 10 base pairs (bp) and maximum mismatch ratio of 0.2 in the overlapping region. It is important to note that solely those merged sequences which satisfied the aforementioned criteria were retained for the purpose of downstream analyses (26).

The utilisation of the UPARSE algorithm facilitated the agglomeration of sequences according to a similarity criterion set at 97%. Subsequently, the removal of chimeric sequences was undertaken, and the importation of the remaining sequences into QIIME2 (version 2022.8) was conducted for the purpose of further processing (27).

The DADA2 algorithm was utilised within QIIME2 to facilitate the quality filtering and denoising of sequence data, thereby ensuring the eradication of any residual chimeric sequences (28). Following a process of data refinement known as ‘de-noising’, a classification of taxonomic origin was then assigned to the representative sequence of each Amplicon Sequence Variant (ASV) by means of the RDP Classifier in conjunction with the SILVA138 reference database. A minimum confidence score of 0.7 was employed for this purpose (29). The number of sequences derived from each sample underwent a standardization process to align with the lowest recorded number of read counts. This was achieved through the random selection of subsets of sequences, considering the inherent variability in the depth of sequencing across different samples. This normalization step ensured the comparability and accuracy of the subsequent downstream analyses.

### 2.13 Statistical analysis

Statistical analysis and the subsequent visualisation of results were conducted utilising the R software (4.1.3 version), incorporating various packages, namely (vegan 2.6–4 version), (phyloseq, 1.38.0 version), (tidyverse, 1.3.2 version), (ggpubr 0.5.0 version), (Complex Heatmap 2.10.0 version), and (corrplot 0.92 version) (30). Alpha diversity metrics – that is to say, diversity indices such as the Simpson (1949) and Shannon (1976) indices, as well as the Sobs index (1948) – were utilised in order to estimate the within-sample diversity. Principal coordinates analysis (PCoA) based on Bray-Curtis’s dissimilarity matrices was performed to visualise the differences in beta diversity between groups. The determination of statistical significance was achieved through the implementation of a permutational multivariate analysis of variance (PERMANOVA) procedure (31). Present study utilised a spearman’s rank correlation analysis to assess the correlations between microbial taxa and other variables of interest (32).

Prior to the analysis, sequences identified as potential chimeras were excluded. To determine the taxonomy of each Operational Taxonomic Unit (OTU) representative sequence, the RDP Classifier was employed against the SILVA138 reference database, with a minimum confidence score set at 0.7. Rarefaction was performed to standardize the sequencing depth across the samples. This process enabled the comparison of operational taxonomic unit (OTU) abundances (29).

### 2.14 Diversity analysis

The circular consensus sequencing (CCS) was employed to generate sequences from the raw data. Subsequent processing steps included barcode identification, length filtering, and chimera removal to obtain high-quality CCS sequences. Subsequent to this, the effective CCS sequences were subjected to clustering and denoising procedures in order to facilitate the classification of operational taxonomic units (OTUs). This analytical approach enabled the identification of microbial communities present in the samples. Subsequent to OTU classification, an evaluation of alpha diversity indices of samples was conducted utilizing QIIME2 software (28). The variation in alpha diversity indices between groups were estimated by using t-test, which enabled the comparison of within-sample diversity across experimental conditions. Beta diversity analysis, which is a method of comparing the degree of similarity in species diversity among different samples, was performed by using QIIME software. The four kinds of algorithms were employed for the analysis of beta diversity: unweighted UniFrac, weighted UniFrac, Bray-Curtis, and binary Jaccard. The utilization of algorithms facilitates acquisition of knowledge regarding the composition and structure of communities. This enables the comparison of microbial diversity between samples based on the presence/absence or abundance of taxa (33).

### 2.15 Species annotation analysis

In order to ascertain statistically significant disparities in microbial composition between vaccine groups and the control group, we undertook an intergroup species abundance analysis in accordance with established protocols (34). The initial taxonomic annotation of feature sequences was executed by employing the SILVA reference database, version 138, with a Bayesian classifier. This approach facilitated species-level assignment, thereby providing a comprehensive framework for taxonomic classification (35). Community composition was quantified at all taxonomic levels (phylum through species) using QIIME2, with visualization of community structure implemented in R (36).

For differential abundance testing, we analyzed species-level data using Metastats software (v2.1) to perform pairwise t-tests between experimental groups. Species contributing to significant compositional differences were filtered based on p-values (p<0.05 considered significant). Due to the large number of microbial subgroups identified, we focused our analysis on the top 10 most significantly differentially abundant species between each vaccine group and control, as presented in **Tables 1**–**4**. This approach follows validated methods for identifying key microbial signatures in vaccine-microbiome studies (34).

**Table 1:** Analysis of differences between groups at CT28 and GV28.

Species	CT28D (mean)	GV28D (mean)	P-value	Variation trend
Beneficial bacterial texa				
*s_uncultured_bacterium_g_eubacterium_siraeum_group*	0.0038971	0.0122638	0.00012	↑
*s_uncultured_bacterium_g_eubacterium_xylanophilum_group*	0.0038569	0.0120328	0.00028	↑
*s_unclassified_g_lachnospiraceae_nk4a136_group*	0.0030634	0.0139010	0.00014	↑
*s_uncultured_bacterium_g_bacteroides_pectinophilus_group*	0.0003415	0.0011551	0.00037	↑
*s_uncultured_bacterium_f_eubacterium_coprostanoligenes_group*	0.0031739	0.0085676	0.00402	↑
*s_uncultured_bacterium_g_roseburia*	0.0032764	0.0003819	0.00321	↑
*s_uncultured_organism_f_muribaculaceae*	0.0018682	0.0106970	0.00777	↑
*s_unclassified_g_monoglobus*	0.0021093	0.0072418	0.00862	↑
*s_uncultured_bacterium_g_eubacterium_ruminantium_group*	0.0068099	0.0130674	0.01734	↑
Pathogenic bacterial texa				
*s_uncultured_bacterium_g_clostridium_sensu_stricto_1*	0.0017778	0.0006328	0.00739	↓
*s_uncultured_bacterium_g_prevotella_9*	0.0434206	0.1213246	0.00176	↓
*s_escherichia_coli*	0.0027722	0.0012656	0.00580	↓
*s_unclassified_g_ruminococcus*	0.0138106	0.0048714	0.01141	↓

P-Value > 0.05, non-significant; P-Value < 0.05, * and P-Value < 0.01, arrows ↑ and ↓ denote increasing and decreasing trends in gut microbial populations, respectively.

**Table 2:** Analysis of differences between CT60D and GV60D.

Species	CT60D (mean)	GV60D (mean)	P-value	Variation trend
Beneficial bacterial texa				
*s_unclassified_g_lachnospiraceae_nk4a136_group*	0.0063680	0.0275007	0.00375	↑
*s_uncultured_bacterium_f_oscillospiraceae*	0.0022800	0.0102048	0.00087	↑
*s_unclassified_f_oscillospiraceae*	0.0054037	0.0109882	0.00308	↑
*s_uncultured_bacterium_g_eubacterium_ruminantium_group*	0.0000402	0.0170649	0.00147	↑
*s_unclassified_g_ucg-005*	0.0015367	0.0073623	0.00159	↑
*s_uncultured_bacterium_g_roseburia*	0.0058557	0.0193349	0.00163	↑
*s_unclassified_f_lachnospiraceae*	0.0434206	0.1273089	0.00209	↑
*s_uncultured_organism_g_butyrivibrio*	0.0204096	0.0002009	0.00248	↓
Pathogenic bacterial texa				
*s_uncultured_bacterium_g_clostridium_sensu_stricto_1*	0.0055343	0.0016271	0.00256	↓
*s_unclassified_f_muribaculaceae*	0.0378361	0.0197969	0.00015	↓
*s_uncultured_bacterium_g_prevotella_9*	0.0041482	0.0376955	0.00059	↓
*s_helicobacter_typhlonius*	0.0277418	0.0041583	0.00031	↓

P-Value > 0.05, non-significant; P-Value < 0.05, * and P-Value < 0.01, arrows ↑ and ↓ denote increasing and decreasing trends in gut microbial populations, respectively.

**Table 3:** Analysis of differences between CT28D and LV28D.

Species	CT28D (mean)	LV28D (mean)	P-value	Variation trend
Beneficial bacterial texa				
*s_unclassified_g_alloprevotella*	0.0020390	0.0211026	0.01972	↑
*s_uncultured_organism_g_butyrivibrio*	0.0018180	0.0048915	0.02159	↑
*s_unidentified_f_oscillospiraceae*	0.0052731	0.0118621	0.01243	↑
*s_lactobacillus_johnsonii*	0.0029831	0.0082864	0.00261	↑
*s_uncultured_organism_f_muribaculaceae*	0.0018682	0.0123341	0.00342	↑
*s_unclassified_g_lachnospiraceae_nk4a136_group*	0.0030634	0.0074628	0.01710	↑
*s_unclassified_g_ruminococcus*	0.0138106	0.0030735	0.00666	↓
*s_uncultured_bacterium_g_eubacterium_xylanophilum_group*	0.0038569	0.0009542	0.00174	↓
*s_uncultured_bacterium_g_eubacterium_ruminantium_group*	0.0068099	0.0005524	0.00043	↓
Pathogenic bacterial texa				
*s_unidentified_o_clostridia_ucg-014*	0.0046303	0.0013459	0.01902	↓
*s_uncultured_prokaryote_g_christensenellaceae_r-7_group*	0.0051828	0.0006026	0.00602	↓
*s_uncultured_bacterium_g_eubacterium_siraeum_group*	0.0038971	0.0013560	0.00008	↓

P-Value > 0.05, non-significant; P-Value < 0.05, * and P-Value < 0.01, arrows ↑ and ↓ denote increasing and decreasing trends in gut microbial populations, respectively.

**Table 4:** Analysis of differences between CT60D and LV60D.

Species	CT60D (mean)	LV60D (mean)	*P-*value	Variation trend
Beneficial bacterial texa				
*s_uncultured_bacterium_g_lachnospiraceae_nk4a136_group*	0.0125350	0.0495375	0.00361	↑
*s_unclassified_g_monoglobus*	0.0020490	0.0193449	0.00362	↑
*s_uncultured_bacterium_g_eubacterium_xylanophilum_group*	0.0038971	0.0125250	0.00186	↑
*s_unclassified_f_lachnospiraceae*	0.0434206	0.1214431	0.00195	↑
*s_unclassified_g_lachnospiraceae_nk4a136_group*	0.0063680	0.0557447	0.00234	↑
*s_uncultured_organism_f_muribaculaceae*	0.0043893	0.0015669	0.00305	↓
*s_unclassified_f_muribaculaceae*	0.0378361	0.0181396	0.00220	↓
*s_uncultured_bacterium_f_muribaculaceae*	0.1102239	0.0551019	0.00035	↓
*s_uncultured_bacterium_g_prevotella_9*	0.1178072	0.0020088	0.00133	↓
Pathogenic bacterial texa				
*s_uncultured_bacterium_o_rf39*	0.0002913	0.0095419	0.00241	↑
*s_bacteroides_sartorii*	0.0211328	0.0006227	0.00001	↓

P-Value > 0.05, non-significant; P-Value < 0.05, * and P-Value < 0.01, arrows ↑ and ↓ denote increasing and decreasing trends in gut microbial populations, respectively.

### 2.16 Metastats analysis

The Metastats software with t-test were conducted to analyzed species abundance data from each group. This statistical analysis calculated p-values, which were then utilized to identify species contributing to differences in sample composition between the two groups. In the course of the analysis, species that demonstrated substantial disparities in abundance among the groups were excluded from further consideration. This procedure was implemented to ensure the reliability and validity of results, as designated by obtained p-values. The implementation of this filtration process resulted in the successful identification of microbial taxa that exerted a substantial influence on the composition of samples obtained from two distinct groups. This achievement yielded invaluable insights, providing a comprehensive understanding of the unique microbial signatures present within each group and the identification of potential biomarkers.

### 2.17 Functional predictive analytics

The feature sequences were annotated and predicted using the PICRUSt2 software, which utilized the existing phylogenetic tree for annotation. Functional data was retrieved from the IMG microbial genome database, enabling the implication of functional gene composition of samples. Statistical methods were then used to assess the significance of differences in the abundance of functional categories between samples or subgroups. For samples with more than 20 annotated functional genes, the G-TEST was used, while the Fisher test was applied to samples with fewer than 20 annotated functional genes. Additionally, the statistical significance of differences between groups was assessed using a pairwise t-test, with a p-value less than 0.05 considered statistically significant. The Kyoto Encyclopedia of Genes and Genomes (KEGG) metabolic pathway analysis showed differences in functional gene pathways among microbial communities in the various subgroups. This investigation provided insights into the functional potential of microbial communities and identified key metabolic pathways associated with differences between samples or groups.

### 2.18 Data analysis

In this study data was analyzed and presented using GraphPad Prism 9.0, with results expressed as mean ± standard deviation. Independent samples t-tests were used to compare means between two groups, while comparisons involving more than two groups were analyzed using either one-way or two-way analysis of variance (ANOVA), p-value <0.05 was considered statistically significant.

## 3 Results

### 3.1 Immunophenotyping of CD4^+^ and CD8^+^ T cells by flow cytometry assay

**Figure 1:**
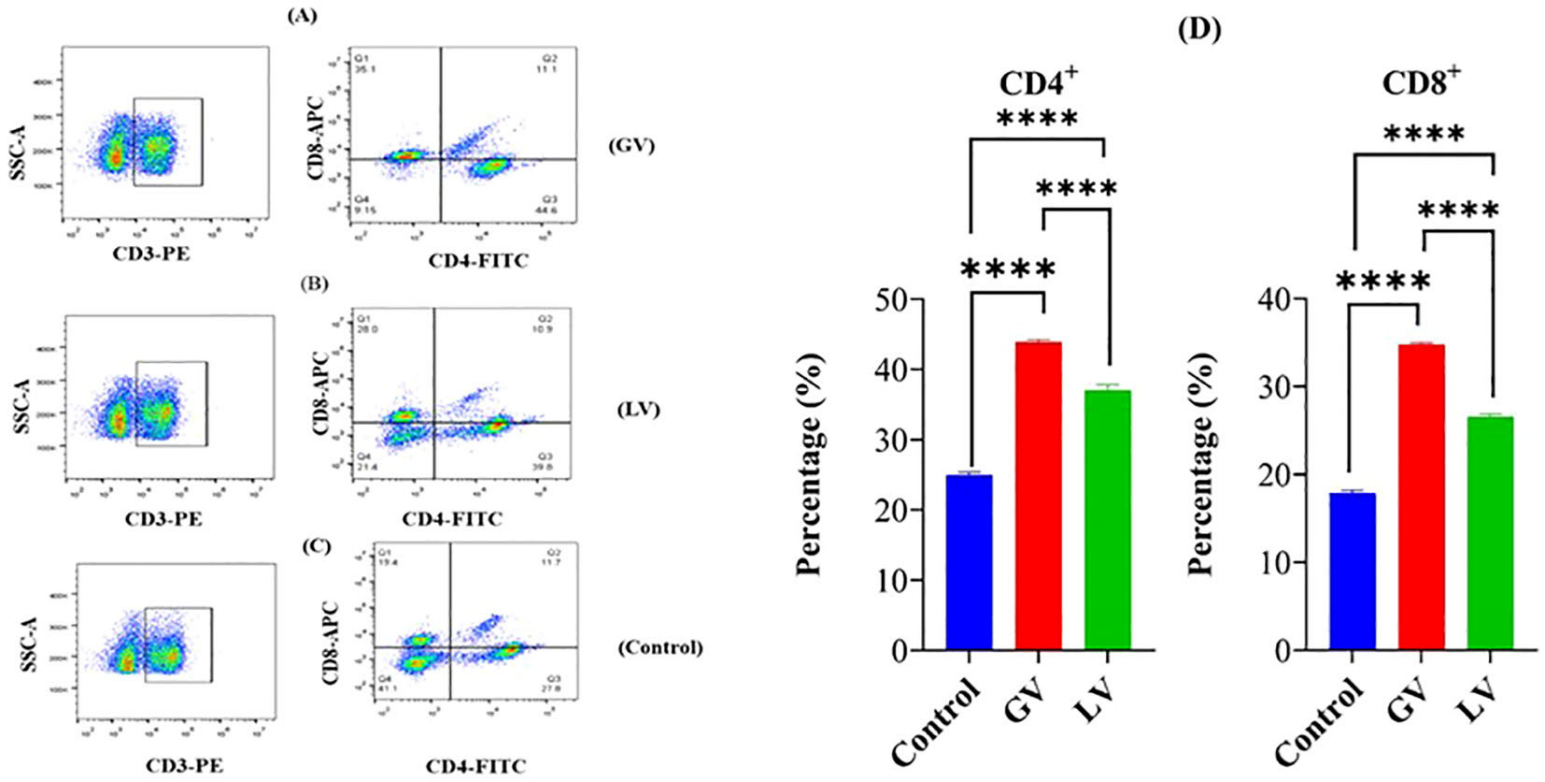
Fluorescence analysis was performed on splenocytes from rats in the GV group **(A)**, LV group **(B)**, and control group **(C)** to assess the expression of CD4^+^ and CD8^+^ markers. The percentage of splenocytes within each defined gate **(D)** is also provided. Spleen cell suspensions from six rats per group were incubated with fluorescein isothiocyanate (FITC)-conjugated anti-rat CD4^+^ monoclonal antibody (mAb), allophycocyanin (APC)-conjugated anti-CD8^+^ mAb, and phycoerythrin (PE)-conjugated anti-rat CD3^+^ mAb. The samples were examined using a flow cytometer (Syntax) and FlowJo software (3-Star, Ashland, OR, USA). The data are represented as percentages of CD4^+^ and CD8^+^ T lymphocytes in the spleens of the experimental rats. Statistical significance is denoted by the number of asterisks (****p < 0.0001).

It has been determined through rigorous analysis that the cell-mediated immune response prompts defensive measures by the host against immunogens, vaccines, viruses, and other pathogens. This is achieved through the promotion of antibody production and the induction of cytotoxic activity. The T-cell subsets (CD4^+^ and CD8^+^) were quantified utilizing an automated cell-counting instrument designed for flow cytometry applications. The outcomes of this quantitative assessment demonstrated that the group receiving the GV intervention elicited a heightened cellular immune response, as evidenced by the augmented percentage of CD4^+^ and CD8^+^ lymphocytes when compared to both the LV group and the control group (p < 0.05; see **Figures 1A-D**).

### 3.2 Assessment of humoral immune response among GV group, LV group and control groups

**Figure 2:**
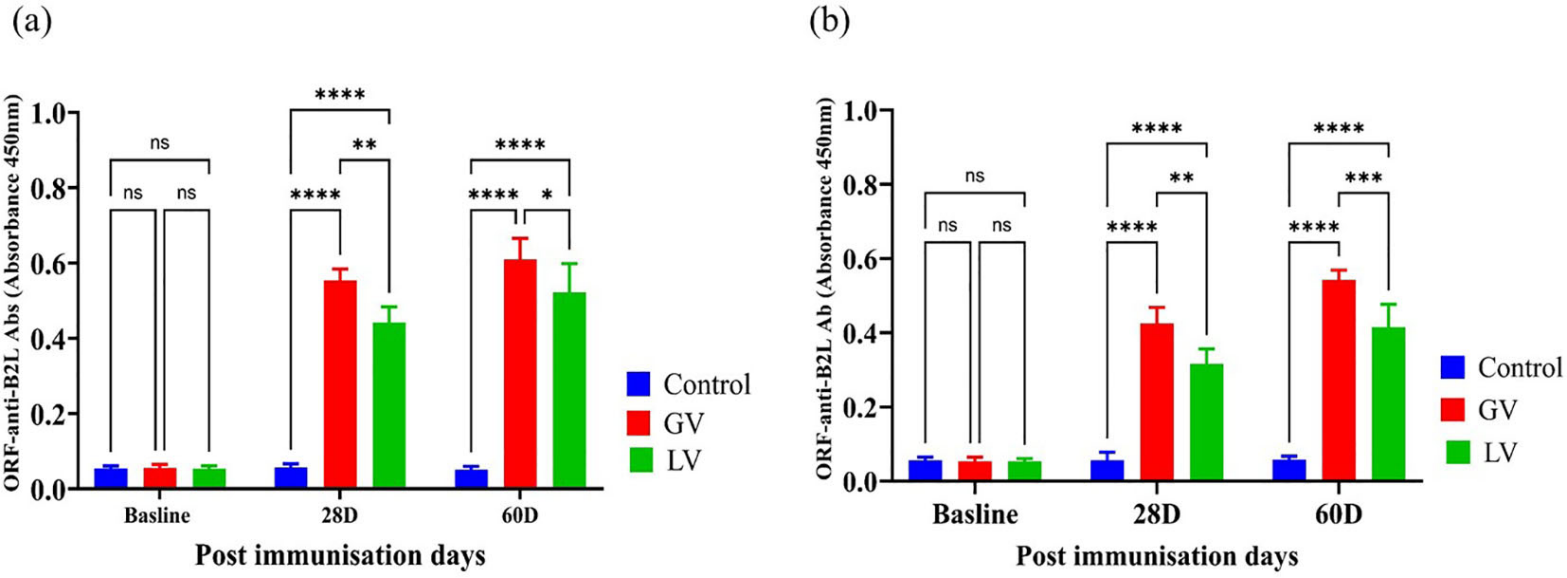
Serum and gut contents ORF Anti-B2L Antibody Response (n=5 per group). **(a)** The ORFV Anti-B2L antibody levels were subsequently measured in serum samples obtained from the subjects at the baseline, 28 days post-immunization (28D), and 60 days post-immunization (60D). **(b)** The ORFV Anti-B2L antibody levels measured in gut contents at the same time points. The data are expressed as mean absorbance values (OD 450nm) ± standard deviation. The statistical significance was estimated using a p-value cutoff of <0.05, with “ns” indicating no significant difference. Asterisks denote with degree of significance as follows: *p < 0.05, **p < 0.01, ***p < 0.001, and ****p < 0.0001.The graph highlights the comparative immune responses in control (blue), GV (red), and LV (green) groups across different post-immunization days.

The serum and gut contents ORF anti-B2L antibody levels were estimated by ELISA before and after immunization. The results demonstrated that the GV group induced a markedly elevated anti-B2L antibody response, at 28 and 60 post immunization com-pared to the LV and control group that was statistically significant as indicated in (p<0.05; **Figures 2a, b**).

### 3.3 Determination of immunoglobulin isotypes post immunization

**Figure 3:**
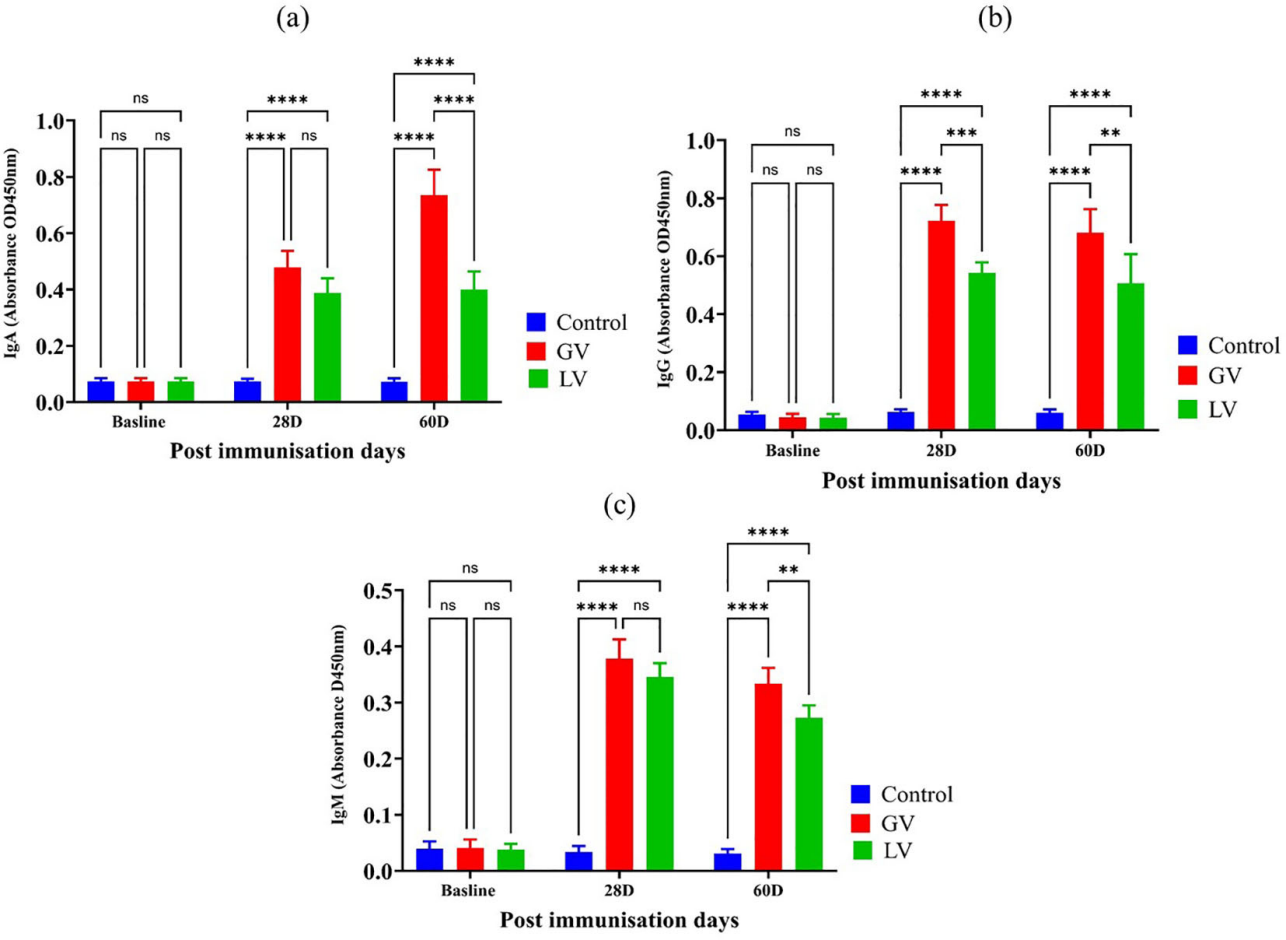
Estimation of Serum Immunoglobulin Isotypes Post-Immunization (n=5 per group). **(a)** The immunoglobulin A (IgA), immunoglobulin G (IgG), and immunoglobulin M (IgM) levels were demonstrated for each corresponding immunoglobulin isotype. The data is expressed as the mean (O.D.) value with standard deviation, providing a graphical representation of the immune response over time at baseline, 28 days post-immunization (28D), and 60 days post-immunization (60D). Statistical analysis was performed with a p < 0.05 threshold to determine the significance of differences between groups. The asterisks symbolize statistical significance, with “ns” indicating no significant difference, and the number of asterisks reflecting degree of significance (*p < 0.05, **p < 0.01, ***p < 0.001, ****p < 0.0001).

To evaluate the humoral immune responses elicited by the vaccination strategies, we quantified serum levels of IgA, IgG, and IgM at baseline and at 28 and 60 days post-immunization. As depicted in **Figure 3b**, at baseline, no significant differences were observed across groups for any immunoglobulin isotype. At 28 day, a significant increase in IgG level was evident in both vaccinated groups compared to the control, with GV group demonstrating a markedly higher IgG response than the LV group (p < 0.001), indicating robust systemic immunogenicity and early class switching. This elevated IgG response was sustained through day 60, with the GV group maintaining significantly higher IgG levels than both the LV and control groups (p < 0.01 and p < 0.0001, respectively). In contrast, IgA and IgM levels showed a distinct kinetic profile. As reflected by IgA levels in **Figure 3a**, at day 28, IgA levels were significantly increased in the GV group compared to both LV and control groups (p < 0.0001), suggestive of mucosal immune priming. This IgA response was further enhanced at day 60, where GV animals exhibited consistently elevated IgA levels relative to both comparator groups (p < 0.0001), highlighting the capacity of the GV formulation to induce durable mucosal immunity. The IgM response, presented in **Figure 3c**, IgM levels also rose significantly in the GV group at day 28 (p < 0.0001), reflective of a strong primary response. Interestingly, IgM levels remained elevated at day 60 in the GV group, surpassing those in the LV and control groups (p < 0.01 and p < 0.0001, respectively), suggesting either prolonged antigen exposure or delayed class-switch recombination potentially modulated by gut microbiota dynamics.

Collectively, these results demonstrate that the GV vaccine elicits a stronger and more sustained immunoglobulin response across all major isotypes, with a distinctive kinetic profile. The early and durable IgG response underscores effective systemic immunity, while the delayed but persistent IgA and IgM elevations suggest an extended phase of mucosal and innate-like humoral activation. These findings highlight not only the immunological strength of the GV formulation but also its potential interactions with the gut microbiota in shaping long-term immune outcomes.

### 3.4 Determination of serum interleukin levels post-immunization at distinct time points

**Figure 4:**
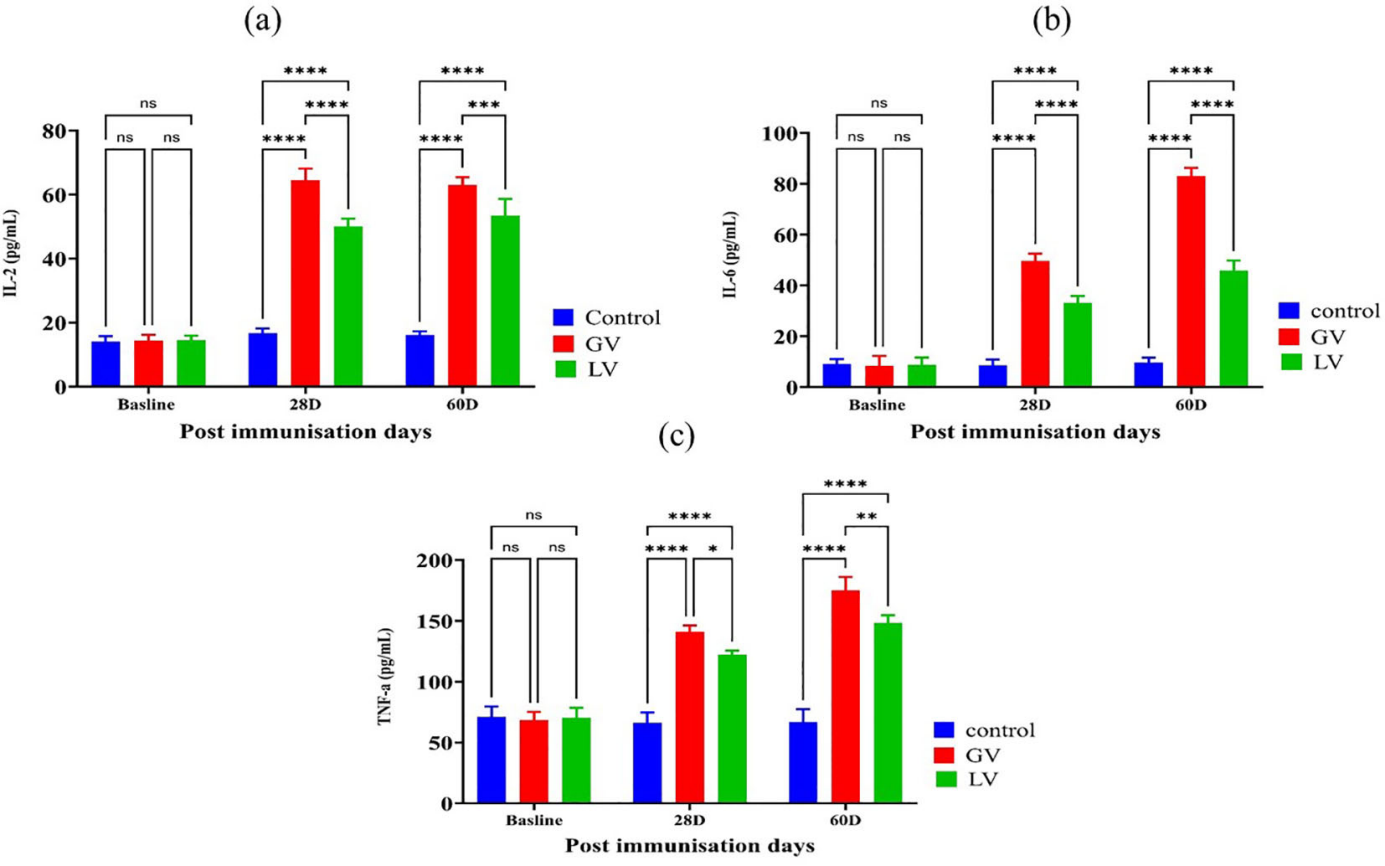
Assessment of interleukin levels in rat serum post immunization (n=5 per group). As indicated by the literature, the levels of interleukins are generally presented through bar graphs for three cytokines: **(a)** interleukin-2 (IL-2), **(b)** interleukin-6 (IL-6), and **(c)** tumor necrosis factor-alpha (TNF-α). The data is presented as the mean ± standard deviation. Statistical significance, as indicated by asterisks, reflects the observed differences in the data. Specifically, the number of asterisks indicates the level of statistical significance, where *p < 0.05, **p < 0.01, ***p < 0.01, ****p < 0.001, and *****p < 0.0001 denote the significance levels, respectively.

In addition, the concentrations of pro-inflammatory interleukins IL-2, IL-6, and TNF-α were evaluated using enzyme-linked immunosorbent assay (ELISA) in the serum of experimental rats prior to and following immunization. The findings indicated that the levels of IL-2, IL-6, and TNF-α were considerably elevated in the GV group compared to the other groups (p < 0.05; **Figures 4a–c**).

### 3.5 Assessment of oxidative status in rat serum post immunization

**Figure 5:**
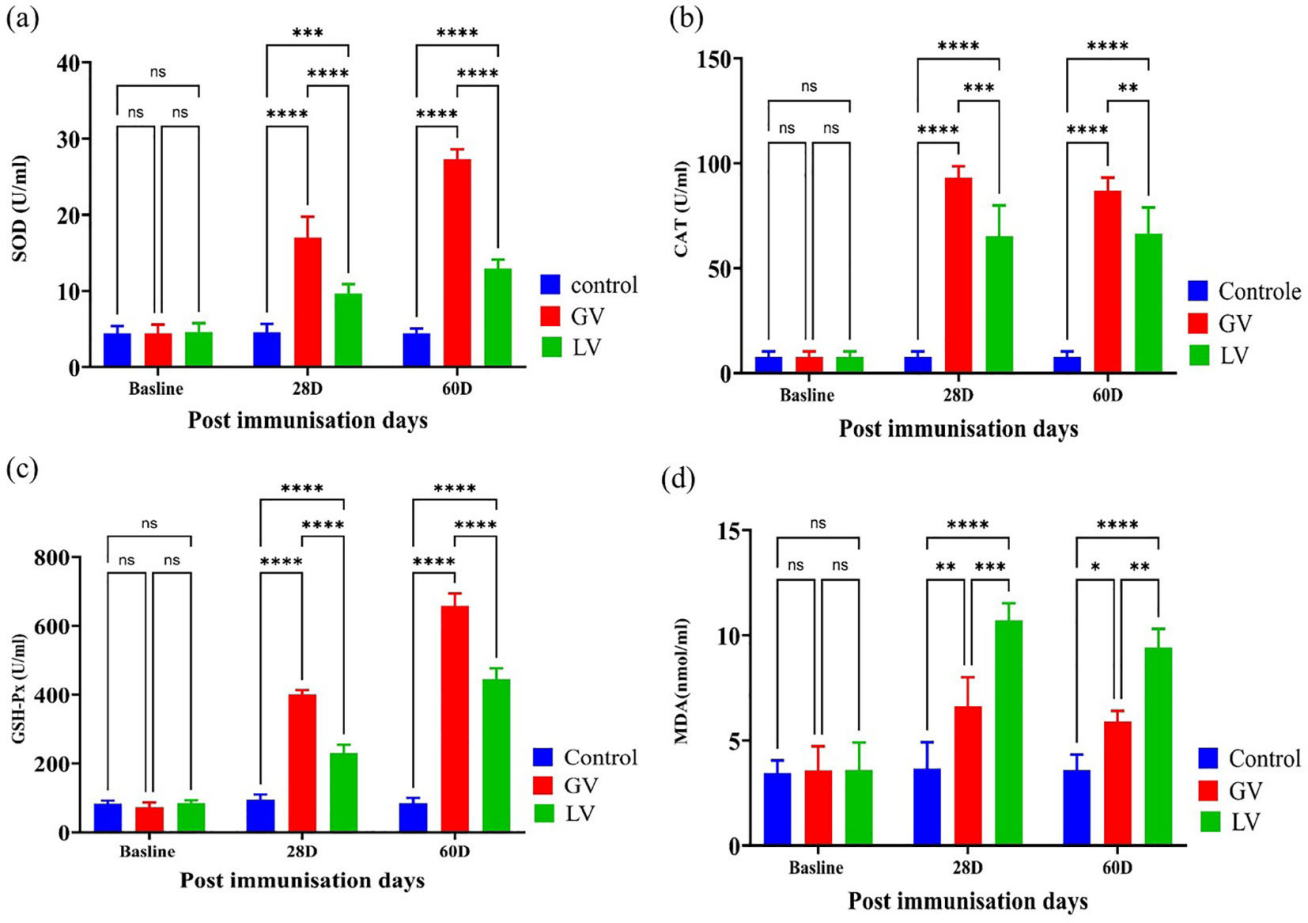
Assessment of Oxidative Status in rat serum post immunization (n=5 per group). The assessment of serum oxidative status was conducted by means of an enzyme activity kit. This evaluation was performed among the GV, LV, and control groups at varying timepoints following immunization. The levels of catalase (CAT), superoxide dismutase (SOD), glutathione peroxidase (GPX), and malondialdehyde (MDA) were measured in the serum over the course of the experiment on different days. The results are presented as mean ± standard deviation. The statistical significance was determined using a p-value threshold of 0.05, with “ns” indicating no significant difference, and asterisks designating the level of significance (*p < 0.05, **p < 0.01, ***p < 0.001, ****p < 0.0001).

In this study, we sought to ascertain the oxidative status in rat serum. To this end, we employed the enzyme-linked immunosorbent assay (ELISA) to measure the oxidative status in rat serum before and after immunization. The findings indicated that the levels of catalase (CAT), superoxide dismutase (SOD), and glutathione peroxidase (GPx) were considerably elevated in the GV group in comparison to the other groups (p < 0.05; **Figures 5a–c**). In contrast, the level of malondialdehyde (MDA) was found to be considerably elevated in the LV group in comparison to the GV group (p < 0.05; **Figure 5d**).

### 3.6 Determination of secretory immunoglobulin in different gut section

**Figure 6:**
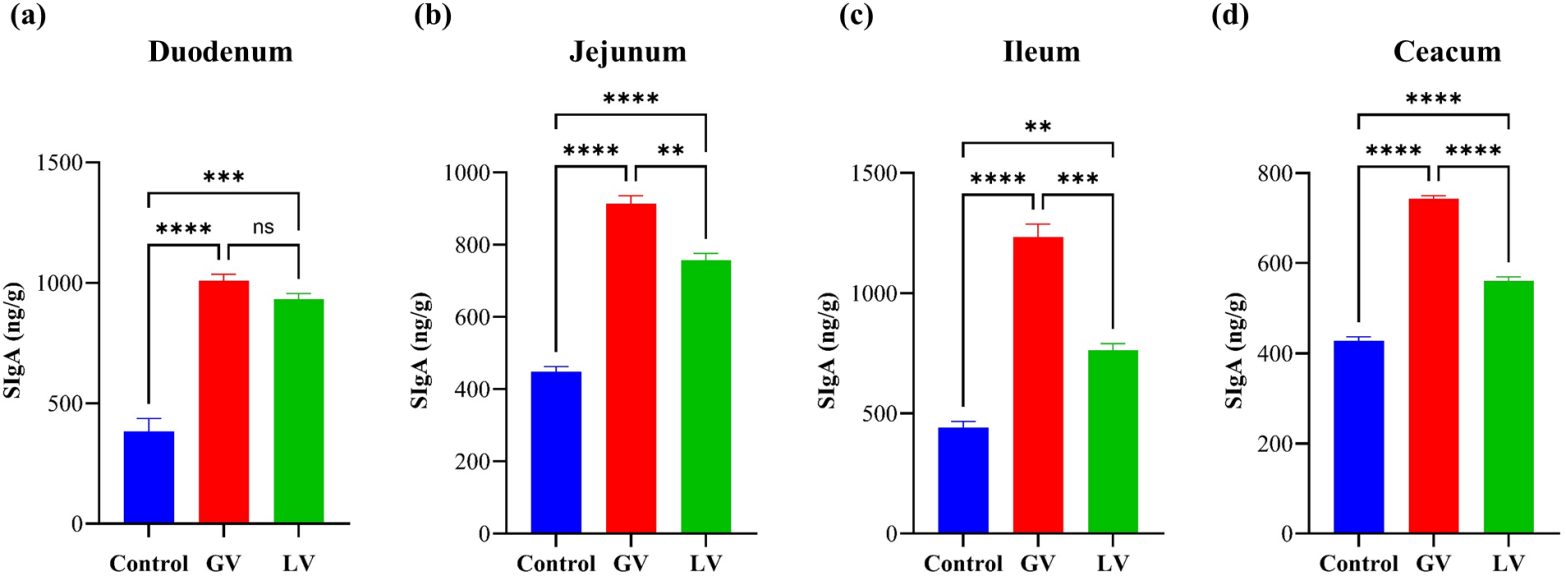
Determination of Secretory immunoglobulin (sIgA) in different gut section (n=5 per group). The bar graphs representing the levels of Secretory Immunoglobulin A (sIgA) in four different parts of the gastrointestinal tract **(a)** Duodenum, **(b)** Jejunum, **(c)** Ileum, and **(d)** Caecum. The sIgA levels are (ng/g). This increase was statistically significant, as indicated by the respective p-values *p<0.05, **p<0.01, ***p<0.001, ****p<0.0001, ns indicating no significant difference.

In the present study, the levels of secretory immunoglobulin A (sIgA) produced were measured in the vaccinated groups and a control cohort using an enzyme-linked immunosorbant assay (ELISA). The findings demonstrated that the GV induced a significantly elevated sIgA level across the jejunum, ileum, and caecum compared to LV and control group, as depicted in (**Figures 6b-d**). Nevertheless, a conspicuous disparity in sIgA levels was not detected between the duodenum and the GV and LV groups, as demonstrated in **Figure 6a**. The observed increase or decrease in the dependent variable was statistically significant, as indicated by the respective p-values *p<0.05, **p<0.01, ***p<0.001, ****p<0.0001.

### 3.7 Alterations in in gut bacterial diversity of rat post immunization

**Figure 7:**
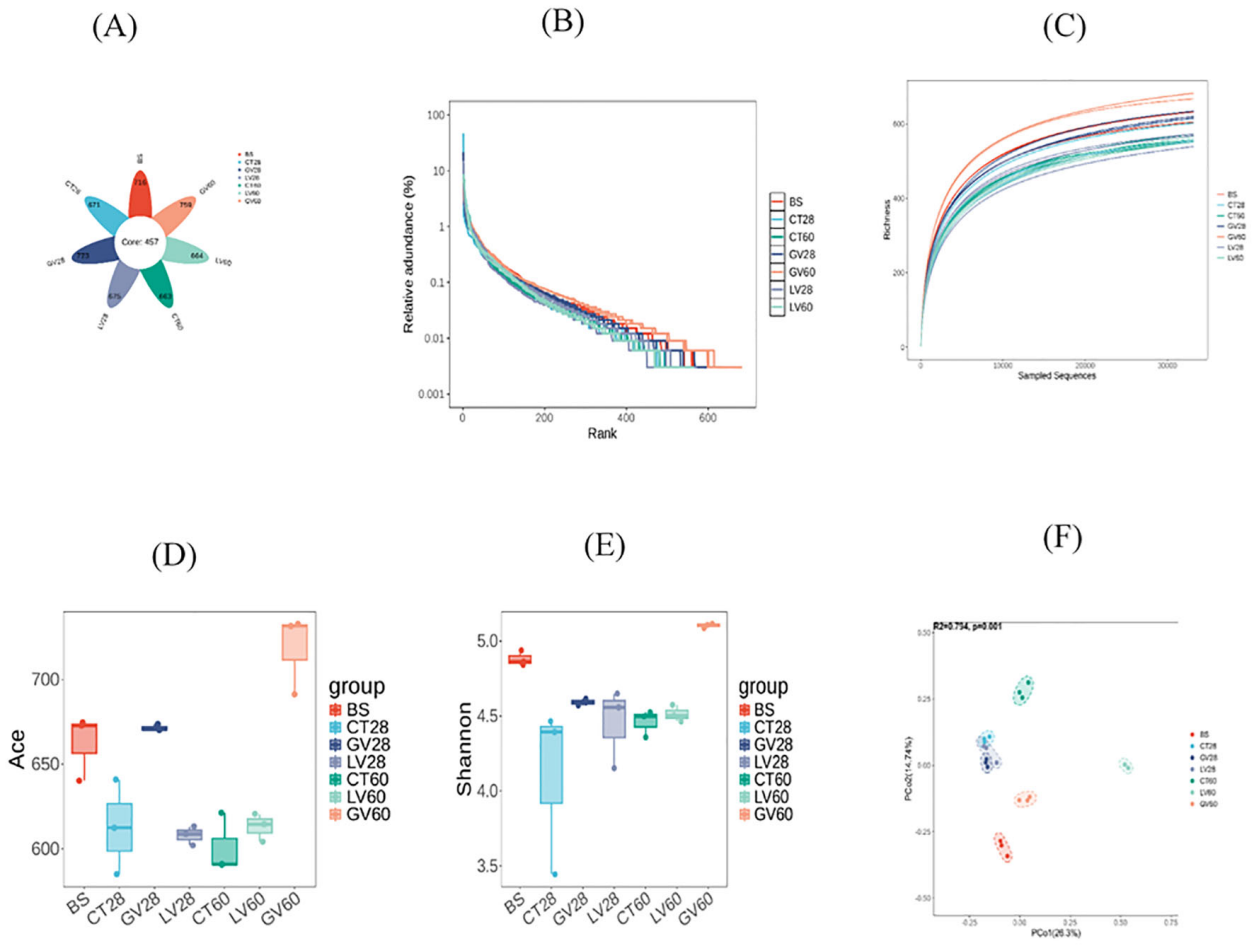
This study explores changes in the diversity of intestinal microbiota in rats before and after immunization with various vaccines, analyzed at two time points: the effector phase (0–28 days) and the memory phase (28–60 days). **(A)** displays a Venn diagram illustrating the intestinal microbiota across different rat groups, derived from operational taxonomic unit (OTU) clustering. The petal diagram provides a visual representation of the Venn diagram, with values in the central area indicating shared characteristics among all samples, while those on the outer edges represent traits unique to each group. In part **(B)** of the experiment, dilution curves for each sample group were generated to evaluate whether the sequencing depth was sufficient to accurately capture species diversity. A steady increase followed by a plateau in the curve suggests that the data volume is adequate for robust analysis. **(C)** presents the cumulative species curve, showing the relationship between the number of samples and the species annotated. Once the curve flattens, it signifies species saturation in the environment, allowing for further analysis. **(D, E)** ACE indices are employed to quantify species abundance and diversity respectively. **(F)** illustrates the results of principal coordinates analysis (PCoA), which was used to assess variations in species diversity across seven sample groups. Each dot represents a sample, with distinct colors indicating different groups. The horizontal and vertical axes correspond to the two principal components accounting for the greatest variance between the samples, with the percentage of impact shown. ACE stands for the Abundance-based Coverage Estimator.

The configuration of caecal microbiota was investigated by 16S rRNA gene sequencing. The total of 13 phyla, 174 differential bacterial genera, and 299 species were identified among rats of three groups. In this amplicon sequencing, 21 samples obtained from experimental rats were performed high-throughput sequencing, and a total of 1636276 sequences were achieved from the three groups. After the sequencing filtering, 1366458 numbers of reads were acquired, with an average of 65069 reads per sample (55362-78105). Intestinal samples contained almost all bacterial species based on the rank abundance and rarefaction curves. The total taxonomic units (OTUs) increased horizontally as the number of samples increased. In total, 4871 OTUs were identified, including 457 common OTUs. The specific OTUs included 716 for the BS group, 671 for the control group at 28 days (CT-28D), 723 for the GV-28D group, 675 for the LV-28D group, 663 for the CT-60D group, 759 for the GV-60D group, and 664 for the LV-60D group, as shown in (**Figure 7A**). Species richness in each sample was assessed through the construction of a rarefaction curve. This curve is generated by randomly subsampling a predefined number of sequences from each sample, determining the number of species represented within those sequences, and then plotting the cumulative number of species against the total number of sequences. The goal of this method is to determine whether the sequencing data is sufficient to accurately represent the species diversity in the sample and provide an indirect measure of the species abundance. The findings of this study indicated that the data set was sufficiently large and varied enough to support the execution of subsequent analyses. This was evidenced by the gradual rise and subsequent leveling off of the curve (**Figure 7B**). Furthermore, the species count curves demonstrated a gradual upward trend and subsequently stabilized at the conclusion of the experiment, indicating number of species in the samples is sufficient to support additional experimental procedures (**Figure 7C**). Alpha diversity is a significant metric for evaluating microbial diversity, particularly in contexts where the habitat is locally homogeneous. This metric reflects both species richness and the evenness of their distribution across individuals within a community, based on samples collected from one or more locations. Common indices used to quantify alpha diversity include the Chao1, abundance-based coverage estimator (ACE), Shannon, and Simpson indices, among others. The Chao1 and ACE indices are used to calculate species abundance, while the Shannon and Simpson indices are used to calculate species diversity.

In this study, the abundance-based coverage estimator (ACE) and Simpson index were used to evaluate the abundance and diversity of rats’ gut microbiota. Additionally, Good’s coverage was calculated to determine the sequencing depth of the samples. Analysis of Good’s coverage values across the six groups resulted in a value of 1, indicating sequencing depth met the requirements for subsequent experiments (**Table 5**). The ACE index was utilized to measure the biodiversity within the sample, with higher ACE values reflecting greater species richness. Notably, the ACE index of the GV-28D group showed a significant increase compared to all other groups (**Figure 7D**). The Simpson index was used to estimate species diversity, with higher values indicate lower diversity. The diversity of the GV-60D group was considerably lower compared to other groups, while CT-28D group exhibited significantly higher diversity (**Figure 7E**). To explore differences in microbial community composition between samples and groups, Beta diversity analysis was performed. Principal coordinate analysis revealed significant structural differences among seven sample groups, with horizontal and vertical coordinates accounting for 26.3% and 14.74% of the variation, respectively (**Figure 7F**). These findings suggest notable differences in microbial composition across the sample groups.

**Table 5:** Comparison of alpha diversity.

Sample	ACE	Simpson	Good’s coverage
BS (0-7D)	662.56 ± 19.32	0.979 ± 0.002	1
GV-28D	671.43 ± 2.06	0.950 ± 0.003	1
GV-60D	718.64 ± 23.61	0.986 ± 0.001	1
LV-28D	608.10 ± 5.54	0.961 ± 0.010	1
LV-60D	613.16 ± 8.35	0.972 ± 0.002	1
CT-28D	612.83 ± 27.96	0.892 ± 0.098	1
CT-60D	601.01 ± 17.68	0.967 ± 0.008	1

The abundance and diversity of the rat’s intestinal flora were evaluated using the ACE and Simpson indices, respectively. Additionally, Good’s Coverage values were utilized to show the sequencing depth of samples. The acronym “ACE” is an abbreviation for “abundance-based coverage estimator.” The baseline BS (0-7D) is the baseline before immunization. The following vaccines were utilized in the study: the GV-28D (Gene vaccine at 28D), the GV-60D (Gene vaccine at 60D), the LV-28D (Live vaccine at 28D), the LV-60D (Live vaccine at 60D), and the CT-28D (Control Group at 28D). The CT-60D (Control Group at 60D) served as the control group.

### 3.8 Species annotation results

**Figure 8:**
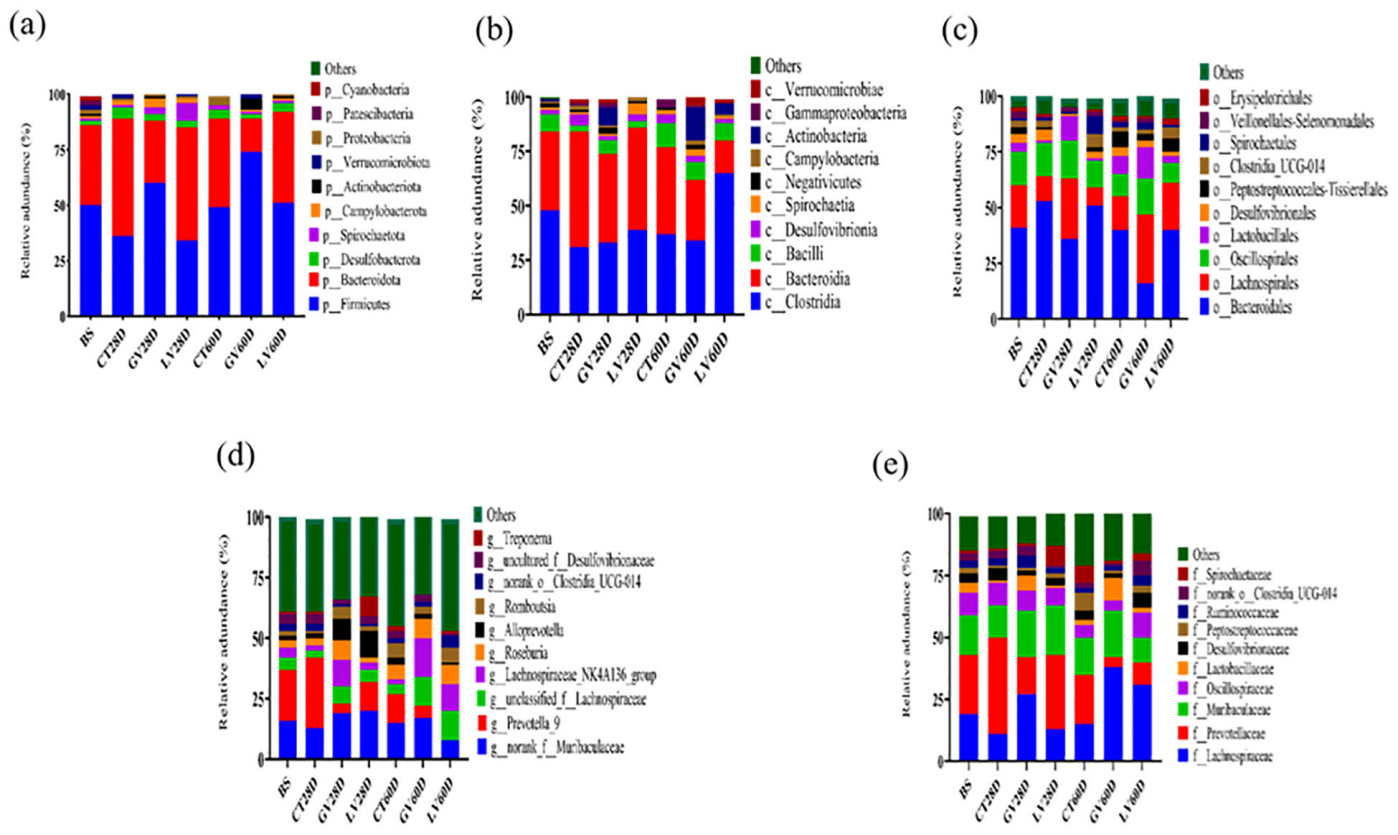
A study was conducted to ascertain taxonomic composition of gut microbiota in rats. Following taxonomic classification system is employed for the purpose of analyzing the composition of gut bacteria: phylum **(a)**, class **(b)**, order **(c)**, genus **(d)**, and family **(e)**.

In this study, we comprehensively analyzed the relative abundance of bacterial taxa at multiple hierarchical levels across distinct time points following immunization to evaluate the immunomodulatory effects on the gut microbiota. **Figure 8** illustrates these compositional shifts, detailing microbial profiles at the **(a)** phylum, **(b)** class, **(c)** order, **(d)** genus, **(e)** and family levels, respectively. This multi-layered taxonomic analysis provides critical insights into how immune activation dynamically reshapes the gut microbial landscape. At phylum level (**Figure 8a**). At phylum level composition, baseline, the gut microbiome was predominantly composed of *Bacteroidota* (53%) and *Firmicutes* (36%), with minor contributions (<5% each),from *Desulfobacterota*, *Spirochaetota*, *Campylobacterota*, *Actinobacteriota*, *Proteobacteria*, *Verrucomicrobiota*, and *Patescibacteria*. By the effector phase (28 days post-immunization), the control group exhibited a marked shift, with *Firmicutes* increasing to 58% and *Bacteroidota* decreasing to 36%. This trend was attenuated in the GV group (GV28D), where *Firmicutes* rose to 51% and *Bacteroidota* constituted 41%. In contrast, the LV group (LV28D) maintained a higher relative abundance of *Bacteroidota* (51%) alongside a reduced proportion of *Firmicutes* (34%). At the memory phase (60 days post-immunization), the control group (CT60D) reverted to a more balanced composition (*Firmicutes*: 49%; *Bacteroidota*: 40%). However, the GV group (GV60D) displayed a pronounced dominance of *Firmicutes* (74%) with a sharp decline in *Bacteroidota* (15%). Conversely, the LV group (LV60D) showed an intermediate profile, with *Firmicutes* at 60% and *Bacteroidota* at 28%.

At genus level composition Prior to immunization, the gut microbiota was dominated by *Prevotella_9* (21%) and unclassified *Muribaculaceae* (16%). By the effector phase (28 days post-immunization), distinct compositional shifts emerged across treatment groups. In the control group (CT28D), *Prevotella_9* abundance increased to 29%, whereas unclassified *Muribaculaceae* decreased to 13%, accompanied by reductions in *Lachnospiraceae_NK4A136_group* (2%) and unclassified *Lachnospiraceae* (3%). In contrast, the GV group (GV28D) exhibited a divergent trend, with unclassified *Muribaculaceae* rising to 19% and *Prevotella_9* sharply declining to 4%, alongside a notable expansion of *Lachnospiraceae_NK4A136_group* (11%). The LV group (LV28D) displayed yet another pattern: unclassified *Muribaculaceae* remained highly abundant (20%), while *Prevotella_9* further decreased to 12%. Strikingly, this group also showed a pronounced increase in *Alloprevotella* (11%) and *Treponema* (8%), suggesting a unique immunomodulatory effect on microbial taxa associated with mucosal inflammation and metabolic regulation as shown in **Figure 8d**.

**Figure 9:**
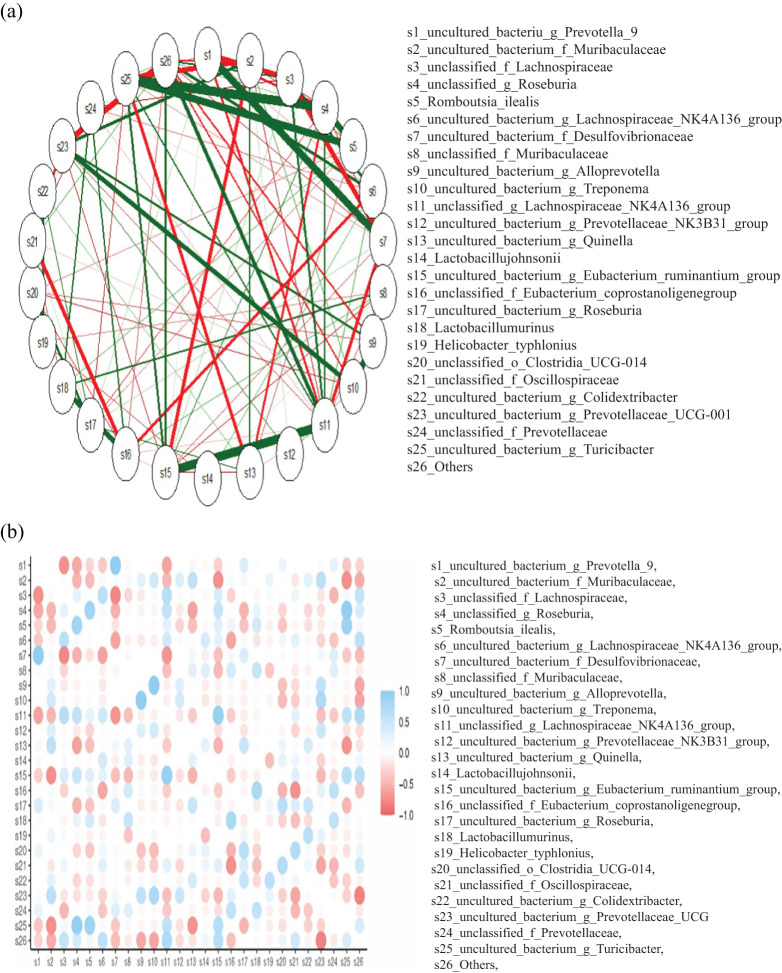
**(a)** The network mapping functions as a correlation analysis tool, assessing the relative abundance of each species within the samples. Variations in these abundances are then examined using Spearman rank correlation analysis. Correlations with a magnitude greater than 0.1 and a p-value below 0.05 are selected to construct a correlation network. The length of the color block indicates the relative abundance of the species, with the circular symbol representing each species, and its size reflecting the average abundance of that species. The lines between species indicate their correlation, with the thickness of the line signifying the strength of the correlation. The color of the line denotes whether the correlation is positive (orange) or negative (green). **(b)** The color gradient ranges from blue, indicative of a positive correlation (approaching +1), to red, denoting a negative correlation (approaching -1), and white, signifying a neutral or zero correlation. The size of the circles within each cell appears to be proportional to the strength of the correlation, with larger circles denoting stronger correlations. The color bar located on the right side of the figure provides a reference range for the correlation values, with 1.0 representing the upper limit of the blue end and -1.0 representing the lower limit of the red end.

To validate these findings, we constructed correlation network mapping diagrams for the top 25 genera, which corroborated the results obtained from the histograms (**Figures 9a, b**). These analyses provided a comprehensive understanding of the microbial taxonomic composition across groups and time points, highlighting dynamic changes in microbial communities following immunization.

### 3.9 Species level analysis of differences between groups

A t-test was performed on species abundance data using Metastats software to calculate p-values, enabling a detailed examination of the variations in the gut microbiota between rats immunized with each vaccine and the control group. The species contributing to the variation in sample composition between two groups were filtered based on their p-values. Given the substantial quantity of identified subtypes, the present study directed its attention toward the execution of inter-group significance analyses at species level. Subsequent presentation will offer a comprehensive overview of the ten most salient groups of data (**Tables 1**-**4**).

The results obtained demonstrated substantial changes in the abundance of specific bacterial species in the GV group in comparison with the control group at the effector time point (28 days post-immunisation, dpi). It is noteworthy that several bacterial taxa exhibited significant increases in abundance, *including unclassified_g_lachnospiraceae_nk4a136_group s_uncultured_bacterium_g_eubacterium_siraeum_group, s_uncultured_bacterium_g_eubacterium_xylanophilum_group*, and *s_uncultured_bacterium_g_roseburia* (p < 0.05). Conversely, other taxa exhibited significant decreases, including *s_uncultured_bacterium_g_clostridium_sensu_stricto_1, s_uncultured_bacterium_g_prevotella_9*, and *s_escherichia_coli* (p < 0.05), as outlined in **Table 1**.

At designated memory time point (60 dpi), substantial alterations in the relative abundance of bacterial species were observed in the GV group as compared to the control group. Specifically, following bacterial taxa exhibited a marked increase in abundance: *s_unclassified_g_lactobacillaceae_nk4a136_group, s_uncultured_bacterium_f_oscillospiraceae, s_unclassified_f_oscillospiraceae, s_uncultured_bacterium_g_eubacterium_ruminantium_group, and s_unclassified_g_ucg-005* (all with p < 0.05). In contrast, the presence of *s_uncultured_bacterium_g_clostridium_sensu_stricto_1, s_uncultured_bacterium_g_prevotella_9*, and *s_helicobacter_typhlonius* was found to be significantly reduced (p < 0.05), as presented in **Table 2**.

At designated effector time point (28 dpi), substantial alterations in bacterial species abundance were detected in the LV group in comparison with the control group. Specifically, a significant increase was found in the abundance of *s_uncultured_organism_f_muribaculaceae, s_unclassified_g_ruminococcus, s_uncultured_bacterium_g_eubacterium_xylanophilum_group*, and *s_lactobacillus_johnsonii* (p < 0.05). Conversely, a marked decrease in the abundance of *s_unclassified_g_ruminococcus, s_uncultured_bacterium_g_eubacterium_xylanophilum_group, and s_unidentified_o_clostridia_ucg-014* (p < 0.05) was observed in the LV group, as outlined in **Table 3**.

As indicated by a significant discrepancy in the abundance of particular bacterial taxa observed between the LV and control groups at the memory time point (60 dpi), it can be posited that the LV treatment may have exerted a notable influence on the composition of the intestinal microbiota. It was found that the bacterial taxa *s_uncultured_bacterium_g_lachnospiraceae_nk4a136_group, s_unclassified_g_monoglobus* and *s_uncultured_bacterium_g_eubacterium_xylanophilum_group* were significantly more abundant in the LV group (p < 0.05). Conversely, the abundance of *s_uncultured_organism_f_muribaculaceae, s_uncultured_bacterium_g_prevotella_9, s_uncultured_bacterium_o_rf39, s_unclassified_f_muribaculaceae*, and *s_bacteroides_sartorii* was significantly lower in the LV group compared to the control group (p < 0.05), as presented in **Table 4**.

### 3.10 Picrust-2 functional prediction

**Figure 10:**
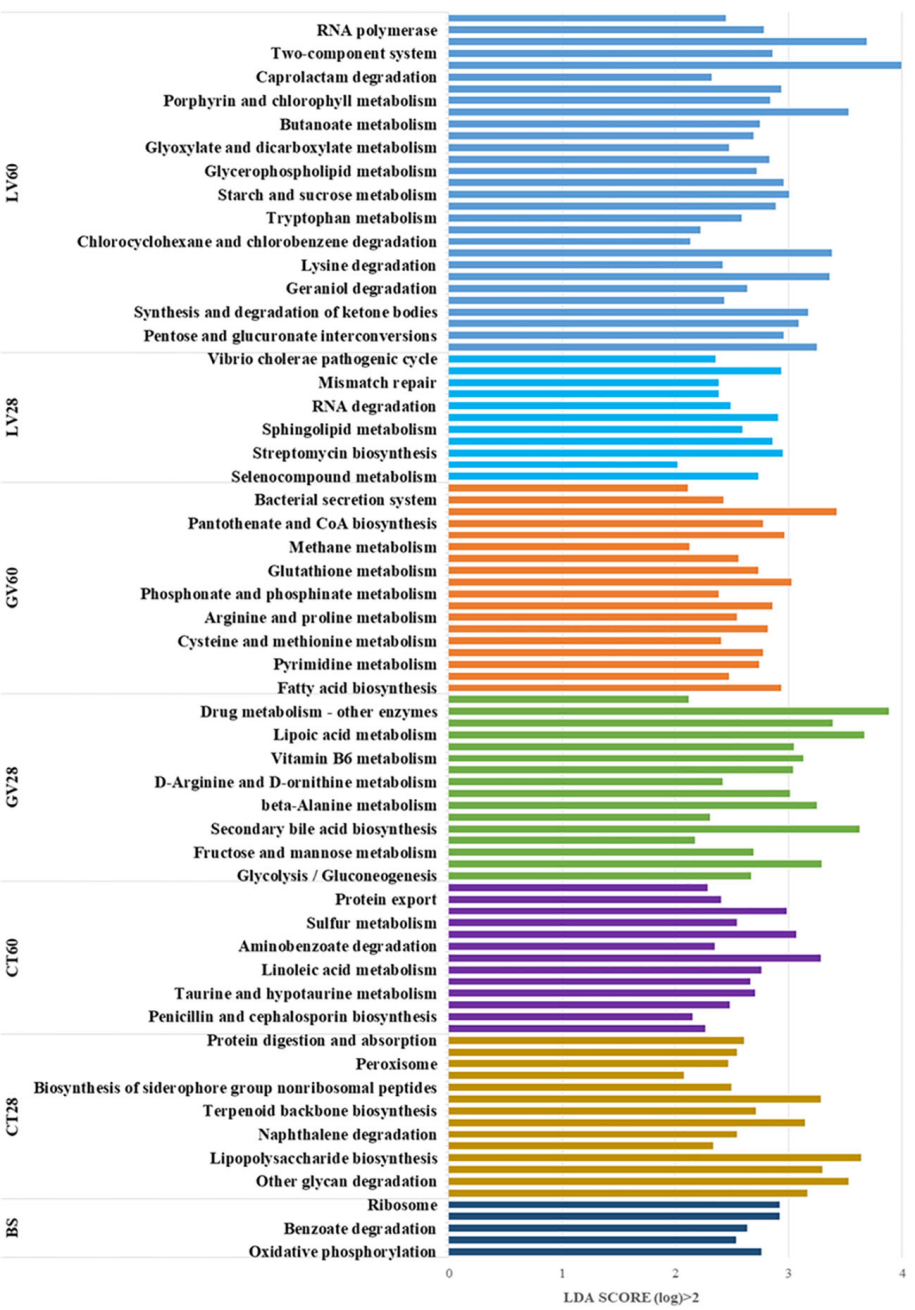
This study examined the enriched KEGG metabolic pathways of the immunised groups in comparison to the control group. The LDA (Linear Discriminant Analysis) score bar chart visualizes the differentially abundant pathways across various groups, with the LDA score indicating the significance of separation between groups. The pathways are sorted based on their LDA scores, which are greater than 2 (log10 scale), highlighting the most significantly different pathways. Below is a group-wise interpretation of the results.

A KEGG metabolic pathway analysis was performed, identifying significant differences in functional genes within gut microbial communities across the sample groups. This approach has been shown to be an effective tool for examining metabolic function modulation in response to environmental variation. The utilisation of Picrust-2 software for the purpose of comparing species composition information obtained from 16S rRNA sequencing data has enabled the identification of enriched KEGG metabolic pathways associated with differential flora. The following metabolic pathways were found to be enriched in the baseline group prior to immunisation: oxidative phosphorylation, phenylalanine metabolism, benzoate degradation, ribosome, and dioxin degradation. In the control group at 28 days post-inoculation (dpi), these metabolic pathways were found to be enriched: ubiquinone and other terpenoid-quinone biosynthesis, other glycan degradation, glycosaminoglycan degradation, lipopolysaccharide biosynthesis, carbon fixation pathways in prokaryotes, tropane, piperidine and pyridine alkaloid biosynthesis. Furthermore, the control group at 60 (dpi) exhibited significant enrichment in the following metabolic pathways: penicillin and cephalosporin biosynthesis, ascorbate and aldarate metabolism, biotin metabolism, protein export, amino sugar and nucleotide sugar metabolism, chloroalkane and chloroalkene degradation, and the phosphotransferase system (PTS). In addition, the presence of Staphylococcus aureus infection metabolic pathways was identified. However, a number of metabolic pathways were found to be significantly enriched in GV group 28 (dpi) sample. These included the citrate cycle (TCA cycle), secondary bile acid biosynthesis, D-glutamine, D-glutamate metabolism, beta-alanine metabolism, riboflavin metabolism, vitamin B6 metabolism, drug metabolism – other enzymes and base excision repair metabolic pathways. In a similar manner, following metabolic pathways were significantly enriched in GV group 60 (dpi): fatty acid biosynthesis, glycine, serine and threonine metabolism, phosphonate and phosphinate metabolism, flagellar assembly, bacterial secretion system and insulin signalling pathway. Furthermore, an analysis of Group 28 (dpi) of LV revealed significant enrichment in several metabolic pathways, including selenocompound metabolism, streptomycin biosynthesis, carbon fixation in photosynthetic organisms, RNA degradation, nucleotide excision repair, mismatch repair, and the cell cycle – Caulobacter, Vibrio cholerae pathogenic cycle pathway. Furthermore, it was observed that Group 60 (dpi) LV exhibited significant enrichment in multiple metabolic pathways, including the pentose phosphate pathway, the synthesis and degradation of ketone bodies, valine, leucine and isoleucine biosynthesis, beta-lactam resistance, C5-branched dicarboxylic acid metabolism, bacterial chemotaxis and the biosynthesis of ansamycins. Consequently, an analysis of the metabolic landscape post-immunisation revealed significant enrichment of specific pathways. The pathways under discussion fall into five distinct categories. Firstly, there are those that are associated with Organismal Systems; secondly, with Metabolism; thirdly, with Cellular Processes; fourthly, with Environmental Information Processing; and fifthly, with Human Diseases. The enrichment was observed at two distinct time points: The designated effector time point is 28 days post-immunization (dpi), while the identified memory time point is 60 dpi. This enrichment was then compared to the pre-immunization state (baseline) and the control group. The differential pathway enrichment is illustrated in **Figure 10**, which presents the outcomes of the Linear Discriminant Analysis (LDA) score bar chart. This method is employed to discern the metabolic pathways that are significantly altered due to the immunization process.

## 4 Discussion

The global persistence of ORFV (Orf virus) in sheep and goat herds highlights the need for improved vaccines. Current attenuated live vaccines are limited by their inability to induce long-lasting immunity and risks such as reversion to virulence, gene deletions, and mutations during cell culture adaptation (37, 38), viral DNA vaccines, however, offer a safer alternative by eliciting both neutralizing antibodies and cellular immune responses [40]. A key feature of ORF vaccines is their ability to activate T-cell-mediated immunity. Our study found that the GV group showed significantly higher percentages of CD4^+^ and CD8^+^ T lymphocytes compared to the LV and control groups (p<0.05; **Figures 1A-D**). CD4^+^ T cells, or helper T cells, play a crucial role in orchestrating immune responses by activating B cells and other immune cells, while CD8^+^ T cells clear virus-infected cells (39). These results align with previous studies showing that CD4^+^ T cells are primary effectors in reducing ORFV load in infected animals (20). Additionally, recent studies have demonstrated that ORFV DNA vaccines elicit strong neutralizing IgG and T-cell responses (40, 41). Our study found that the GV group induced a significantly elevated anti-B2L antibody response at 28 and 60 days post-immunization (dpi). Additionally, the GV group showed notably higher levels of anti-B2L antibodies in gut con-tents, particularly between 28 to 60 dpi, compared to the LV and control groups. This suggests that the GV vaccine effectively stimulates gut mucosal immunity, which is crucial for protective immunity. These results align with previous studies showing that ORF DNA and subunit vaccines elicit strong humoral responses, while attenuated vaccines of-ten have poor immunogenicity (20, 42, 43). However, a live attenuated Orf-V D1701 strain vaccine was reported to protect ewes for 4–6 months (44). Differences in outcomes may stem from variations in ORFV strains, physiological stages, and animal models. Vaccines administered via mucosal routes can induce both mucosal and systemic immune responses, whereas parenteral vaccines primarily elicit humoral immunity (45).

The study found that the GV group significantly elevated IgA and IgG antibody levels compared to the LV and control groups post-immunization. While IgM levels showed no significant difference among vaccinated groups at 28 days post-immunization (dpi), the GV group exhibited significantly higher IgM levels at 60 dpi. These findings align with previous research showing that mRNA vaccines boost IgG and IgA levels in individuals exposed to SARS-CoV-2 (46). The immune response to SARS-CoV-2 typically involves IgM appearing around day 4, peaking by day 20, and fading, while IgG emerges in the first week and peaks around one month (47). The presence of IgG and IgA months post-infection indicates a sustained humoral immune response, with IgA often appearing earlier than IgM (48). This suggests a robust, multi-clonal B cell response post-vaccination. Cytokines like IL-2, IL-4, IL-6, and TNF-α play a vital role in post-vaccination immune responses by activating T cells, guiding their differentiation, and enhancing antibody production (49). Studies, including (50), show that vaccines, such as ORF DNA and Pfiz-er-BioNTech mRNA (BNT162b2), significantly increase levels of cytokines, IL-2, IL-6, and TNF-α, which are crucial for T and B cell activation, neutralizing antibody production, and strong antigen-specific responses. IL-4 regulates Th2-mediated immunity, aiding macrophage activation and germinal center formation, while IL-6 stimulates innate immunity and CD8^+^ T cell proliferation. These cytokines balance pro-inflammatory and regulatory responses, ensuring effective and safe vaccine outcomes.

Oxidative stress, an imbalance between oxidant and antioxidant mechanisms, plays a key role in viral infection pathogenesis. Viral replication and inflammation during acute immune activation increase oxidative stress, generating reactive oxygen and nitrogen species (51–53). Vaccination against Influenza and SARS-CoV-2 has also been linked to elevated oxidative stress (54). Immune responses involve macrophage and dendritic cell activation, producing free radicals and inflammation (55). Normally, reactive oxygen species (ROS) are neutralized by antioxidant enzymes like catalase, superoxide dismutase (SOD), and glutathione peroxidase (GPx) (56, 57). In this study, oxidative stress and antioxidant biomarkers (CAT, SOD, GPx, MDA) were analyzed in vaccinated rats. Results showed higher SOD, CAT, and GPx levels in the GV group, while MDA levels were elevated in the LV group. These findings align with studies showing parapoxvirus ovis (iPPVO) induces oxidative burst in monocytes (58), and human neutrophils (59). Increased CAT levels counteract toxic free radicals, preventing cellular damage (60).

Secretory IgA (SIgA), a dimeric immunoglobulin linked by a joining chain (JC) and complexed with a secretory component (SC), protects mucosal surfaces by neutralizing pathogens and preventing epithelial adherence (61, 62). Our study found that the GV significantly increased sIgA levels in the jejunum, ileum, and caecum compared to the LV and control groups, indicating enhanced local mucosal immunity in these regions. However, no significant sIgA elevation was observed in the duodenum, likely due to its unique anatomical and immunological environment, including high digestive enzyme activity and fewer Peyer’s patches (63). SIgA is crucial for mucosal defense, neutralizing pathogens and maintaining gut homeostasis by interacting with commensal microbiota (64, 65). The GV’s ability to boost sIgA in specific gut regions aligns with findings from intranasal vaccines, which also enhance mucosal immunity (66). Additionally, sIgA’s high anti-gen-binding affinity and stability make it effective against SARS-CoV-2, with potential for aerosolized delivery (67, 68). In conclusion, the GV’s differential sIgA induction high-lights the importance of regional immunological variations in the gut. These findings support the use of parenteral vaccines to enhance mucosal immunity and provide targeted pathogen protection. Future research should optimize vaccine formulations to maximize sIgA production across all mucosal surfaces. With advancements in PCR and nucleic acid technologies, 16S rRNA gene detection has become a key tool for bacterial identification (69). While studies have explored the link between gut flora and viral vaccines (14), limited research exists on how vaccination against ORF disease impacts gut microbiota. Vaccination may reduce harmful bacteria, increase beneficial bacteria, and improve over-all health. In this study, we sequenced 16S rRNA genes from rats immunized with GV and LV to provide insights into vaccine effects on gut flora and support ORF disease vaccine research.

Gut microbiome plays a pivotal role in modulating host immune responses, and our study demonstrates that immunization significantly alters gut microbial composition at both the phylum and genus levels. These shifts were time-dependent and varied across treatment groups, suggesting distinct immunomodulatory mechanisms influenced by vaccine type. At baseline, the predominance of *Bacteroidota* (53%) over *Firmicutes* (36%) aligns with typical gut microbiota profiles in healthy individuals, where these phyla collectively constitute 90% of the bacterial population (34). The effector phase (28 days post-immunization) revealed a marked increase in Firmicutes (58%) in controls, consistent with studies linking *Firmicutes* enrichment to pro-inflammatory immune activation (70). The attenuated shift in the GV group (*Firmicutes*: 51%; *Bacteroidota*: 41%) suggests a milder immunological perturbation, possibly due to adjuvant-driven immune regulation (5). Strikingly, the LV group maintained a *Bacteroidota*-dominant profile (51%), resembling microbiota states associated with enhanced mucosal tolerance (71). By the memory phase (60 days), the GV group exhibited extreme *Firmicutes* dominance (74%), a profile correlated with metabolic dysregulation in prior work (5). In contrast, the LV group’s intermediate *Firmicutes* (60%) and higher *Bacteroidota* (28%) may reflect a stabilized host-microbiome equilibrium, akin to post-vaccination recovery patterns observed with live-attenuated vaccines (72).

At genus level baseline dominance of Prevotella_9 (21%) and unclassified Muribaculaceae (16%) is consistent with their roles in fiber fermentation and immune homeostasis [93]. The control group’s increase in Prevotella_9 (29%) parallels studies associating this genus with Th17-driven inflammation (73), while the decline in Muribaculaceae (13%) may reflect reduced anti-inflammatory signaling (74).

The GV group’s divergent trends expansion of *Lachnospiraceae_NK4A136_group* (11%) and depletion of *Prevotella_9* (4%)—mirror findings where adjuvants promoted short-chain fatty acid (SCFA)-producing bacteria, enhancing regulatory T-cell responses (75). The LV group’s unique rise in Alloprevotella (11%) and Treponema (8%) is particularly intriguing. Alloprevotella has been linked to improved gut barrier integrity (76), while *Treponema*’s increase, though often associated with dysbiosis, may indicate context-dependent roles in mucosal immunity (77). Our results echo vaccine studies reporting *Firmicutes* enrichment during acute immune activation (e.g., influenza vaccination (78), but contrast with others showing *Bacteroidota* stability post-vaccination (e.g., oral polio vaccine (5). These discrepancies may stem from vaccine formulation (e.g., live-attenuated *vs*. subunit), as adjuvants like those in the GV group can differentially recruit immune pathways altering microbial niches (15). The LV group’s Treponema surge, while unusual, finds parallels in microbiome studies of oral vaccines, where transient pathobiont blooms were tied to mucosal IgA induction (79).

The observed shifts suggest that vaccine-induced immunity may transiently trade off microbial diversity for immune efficacy, a phenomenon noted in antibiotic-treated mice reconstituted with vaccine-responsive strains (80).

Our analysis revealed significant alterations in gut microbial composition abundance of specific bacterial species following vaccination, particularly in the GV group at 28 days post-immunization (dpi). Notably, we observed a marked increase in beneficial bacterial species comprising *Lachnospiraceae NK4A136_group*, *Eubacterium siraeum_group*, *Eubacterium xylanophilum_group*, and *Roseburia* (p < 0.05), all of which are known for their metabolic and immunomodulatory functions. Conversely, potentially pathogenic species such as *Clostridium sensu stricto 1*, *Prevotella 9*, and *Escherichia coli* showed significant reductions (*p* < 0.05). These findings align with previous studies demonstrating the critical role of *Lachnospiraceae NK4A136_group* in gut health. Specifically (81),, reported its anti-inflammatory effects in a murine colitis model, where it promoted intestinal mucosal repair. Additionally, this taxon is associated with bile acid metabolism, suggesting a role in cholesterol homeostasis (82). Further supporting our observations, identified *Lachnospiraceae NK4A136_group* as a butyrate producer, a short-chain fatty acid (SCFA) essential for maintaining gut barrier integrity and metabolic health. Its probiotic potential has also been validated in murine models, reinforcing its significance in microbiome-mediated immune modulation (83).

The enrichment of *Eubacterium* and *Roseburia* both recognized butyrate producers further underscores a vaccine-induced shift toward a metabolically favorable microbiota. Butyrate not only serves as an energy source for colonocytes but also exhibits anti-inflammatory properties through regulatory T-cell (Treg) induction (75). Conversely, the decline in *Clostridium sensu stricto 1* (a pathobiont linked to diarrheal diseases) and *E. coli* suggests reduced risk of gut dysbiosis and inflammation post-vaccination. Collectively, these microbial shifts highlight the potential of GV to foster a resilient, health-associated gut ecosystem.

Our findings highlight significant changes in key bacterial species with important functional implications for gut health*. Eubacterium xylanophilum*, a probiotic anaerobic bacterium equipped with flagella for motility (83–85), demonstrated vaccine-associated enrichment. This fiber-degrading specialist preferentially utilizes cellulose, cellobiose, and xylan (85, 86), and its increase following vaccination mirrors observations from HTI vaccine studies where it correlated with enhanced T-cell responses and cytokine production. Notably, both *Eubacterium xylanophilum* and the co-enriched *Roseburia* genus (14), are potent butyrate producers, with wheat bran polysaccharides shown to specifically stimulate Eubacterium xylanophilum-mediated butyrogenesis (87, 88).

In contrast, we observed concerning associations with diarrheal pathogens*. Clostridium sensu stricto 1* and *Bacteroides* abundances correlated positively with diarrhea incidence, consistent with *Clostridium’s* established role in community-acquired diarrheal outbreaks (89). This aligns with clinical reports identifying *Clostridium perfringens and C*. *difficile* as major diarrheal pathogens alongside *Salmonella* spp (90). Particularly noteworthy was the inverse relationship between *Clostridium sensu stricto 1* abundance and lymphocyte/white blood cell counts in piglets (91), suggesting potential immunosuppressive effects of this pathobionts.

Our results demonstrate that LV immunization induces significant remodeling of the gut microbiota at 28 days post-immunization, characterized by two key patterns: enrichment of potentially beneficial taxa including *Muribaculaceae, Eubacterium xylanophilum group*, and *Lactobacillus johnsonii*, coupled with depletion of inflammation-associated species such as *Ruminococcus* and *Clostridia UCG-014* (*p* < 0.05). These findings align with established vaccine-microbiome interactions observed in *E. granulosus* immunization studies, which similarly showed increased *Lactobacillus taiwanensis* and decreased *Ruminococcus bromii* abundance (34). The expansion of *Lactobacillus johnsonii* is particularly noteworthy as it mirrors previous reports of its ability to remodel gut ecosystems by promoting *Muribaculaceae* while suppressing *Clostridia UCG-014* (92), suggesting this may represent a conserved response to vaccination. The reduction in *Ruminococcus* sp*ecies* carries important clinical implications given its established association with Crohn’s disease (93), and its complex, context-dependent relationship with metabolic disorders - while some studies link *Ruminococcus* to type II diabetes, others paradoxically show its increase during diabetes remission (94). Collectively, these microbial shifts suggest LV immunization promotes a gut environment favorable for mucosal health, characterized by enhanced colonization resistance through *Lactobacillus* expansion, reduced inflammatory potential via *Ruminococcus* depletion, and improved metabolic homeostasis mediated by butyrate-producing *Eubacterium.* The consistency between our findings and other vaccine studies (34, 92), points to potentially universal vaccine-microbiome interaction patterns, though further research is obligatory to elucidate precise mechanisms linking these microbial changes to immune outcomes.

At the memory phase (60 dpi), we observed profound and sustained alterations in gut microbial composition following LV immunization, marked by significant enrichment of beneficial taxa including *Eubacterium ruminantium group, Lachnospiraceae NK4A136_group, and Eubacterium xylanophilum group*, alongside depletion of potentially pathogenic species such as *Bacteroides sartorii, Prevotella 9*, and *Muribaculaceae*. These findings align with previous reports demonstrating similar microbial shifts in healthy versus pathological states, including reduced *Eubacterium ruminantium* group and *Prevotella* abundance in missed abortion cases (95). The observed expansion of *Lachnospiraceae-*associated species is particularly noteworthy given their well-established role in gut homeostasis. *Eubacterium ruminantium*, a key member of this family, has been implicated in neuroprotection through modulation of gut-brain axis signaling (96), while its metabolic by-products (particularly short-chain fatty acids like butyrate) exert systemic anti-inflammatory effects relevant to inflammatory bowel diseases, metabolic disorders, and even colorectal cancer (88). The butyrogenic capacity of *Lachnospiraceae* sp*ecies* contributes to gut barrier integrity through multiple mechanisms: serving as the primary energy source for colonocytes, inhibiting NF-κB-mediated inflammation (97), and promoting regulatory T cell differentiation - effects that may extend to neuroprotection as suggested by their ability to mitigate dopaminergic neurodegeneration (98). Interestingly, these vaccine-induced microbial changes mirror observations from HIV-1 DNA vaccine studies showing similar inverse relationships between *Prevotella-9* depletion and robust IgG responses (14), suggesting potential conserved mechanisms of vaccine-microbiome-immune interactions across different vaccine platforms.

This study demonstrates that the GV group exhibited a significant enrichment of beneficial gut microbiota, notably *Lachnospiraceae_NK4A136_group* bacterial species a linked to short-chain fatty acid production and mucosal homeostasis alongside a reduction in potentially pathogenic species such as *Clostridium sensu stricto 1*, which is associated with dysbiosis and inflammatory conditions. These shifts suggest a vaccine-induced modulation of the gut microbiome toward a more immunoprotective composition. However, our findings are limited by the absence of an *in vivo* infection model, which precludes definitive assessment of how vaccine dosage influences microbial dynamics during active disease. Future studies should integrate challenge models to delineate the functional consequences of these microbiota alterations on pathogen susceptibility and immune efficacy.

## 5 Conclusion

This study demonstrates that the ORF genetic vaccine induces a robust and sustained immune response, marked by enhanced cellular and humoral immunity, mucosal protection, and reduced oxidative stress. Notably, the observed modulation of gut micro-biota post-immunization underscores the vaccine’s potential to improve overall health outcomes, highlighting the gut microbiota as a novel and promising avenue for enhancing vaccine immunogenicity. Analysis of the metabolic landscape revealed significant enrichment of specific pathways at two key time points: 28 days post-immunization (dpi), designated as the effector phase, and 60 dpi, identified as the memory phase. These pathways, categorized under Organismal Systems, Metabolism, Cellular Processes, Environmental Information Processing, and Human Diseases, were enriched compared to both the pre-immunization baseline and the control group. These findings suggest that the ORF genetic vaccine not only elicits a strong immune response but also influences metabolic and microbial pathways that contribute to its efficacy. Future research should focus on optimizing vaccine formulations and delivery methods to maximize these benefits while exploring the intricate interactions between gut microbiota and vaccine-induced immune responses. Such insights could pave the way for innovative strategies in vaccine design, ultimately advancing global public health. Collectively, these results position the ORF genetic vaccine as a superior alternative to traditional live attenuated vaccines for combating contagious ecthyma.

## Data Availability

The raw data supporting the conclusions of this article will be made available by the authors, without undue reservation.
